# Estimating the health and economic impacts of Individual Placement and Support (IPS) for people with mental and physical health conditions: A cost-benefit modelling study^☆☆^

**DOI:** 10.1016/j.puhip.2026.100867

**Published:** 2026-10-06

**Authors:** Holly Clarke, Damon Morris, Megan James, Alan Brennan, Adam Whitworth

**Affiliations:** a Sheffield Centre for Health and Related Research (SCHARR), Division of Population Health, School of Medicine and Population Health, University of Sheffield, Sheffield, United Kingdom; b Scottish Centre for Employment Research (SCER), Strathclyde Business School, University of Strathclyde, Glasgow, United Kingdom

**Keywords:** Economic evaluation, Cost–benefit analysis, Individual placement and support, Employment, Mental health, Musculoskeletal conditions, Labour market participation, Health-related economic inactivity

## Abstract

**Objectives:**

We evaluated the effectiveness and long-term economic impacts of IPS for people with mental health (MH), musculoskeletal (MSK), or combined conditions. Using data from recent trials in England, we developed a cost-benefit and outcomes modelling tool (IPS-MOD).

**Study design:**

A decision-analytic Markov state-transition model to track the impact of interventions on employment trajectories and the proportion of people in employed and non-employed states over a five-year horizon.

**Methods:**

IPS-MOD applied a Markov model structure to simulate transitions between employment and non-employment under IPS and business-as-usual. It incorporated trial evidence, national survey data, and tax-benefit microsimulation to estimate employment rates, quality-adjusted life years (QALYs), government expenditure, and net-earnings over five years. Sensitivity analyses tested the robustness of benefit-cost ratios under alternative assumptions about service costs and effectiveness.

**Results:**

For people with MSK conditions, assuming a societal perspective, IPS-MOD estimated that £1 invested in IPS generated a return of £1.13 by Year 1 and £5.71 by Year 5. By Year 5, IPS was estimated to raise employment rates by 5.87% relative, add 0.137 QALYs per person, and reduce spending by £1009.92 in social security payments and £124.59 in health service use. Positive returns were also observed for MH and combined conditions. Sensitivity analyses showed positive returns by Year 5 across most scenarios.

**Conclusions:**

Expanding IPS services to people with MH, MSK, or combined conditions in England is likely to deliver long-term health and economic benefits and provide a positive economic return on investment. The results contribute to evidence on the value of employment support for people with health conditions and may inform future policy decisions on improving labour market participation.

## 1 Introduction

Economic inactivity among working-age adults in the UK has increased in recent years, with long-term ill health a major driver of this trend. [1] Mental health and musculoskeletal conditions account for a substantial share of health-related inactivity, underlining their central role in shaping labour market participation. [2,3] Although not all inactive individuals are able to return to work, many report that they would like to do so, with sickness and disability representing leading barriers [1,4] (see Fig. 1).

**Fig. 1:**
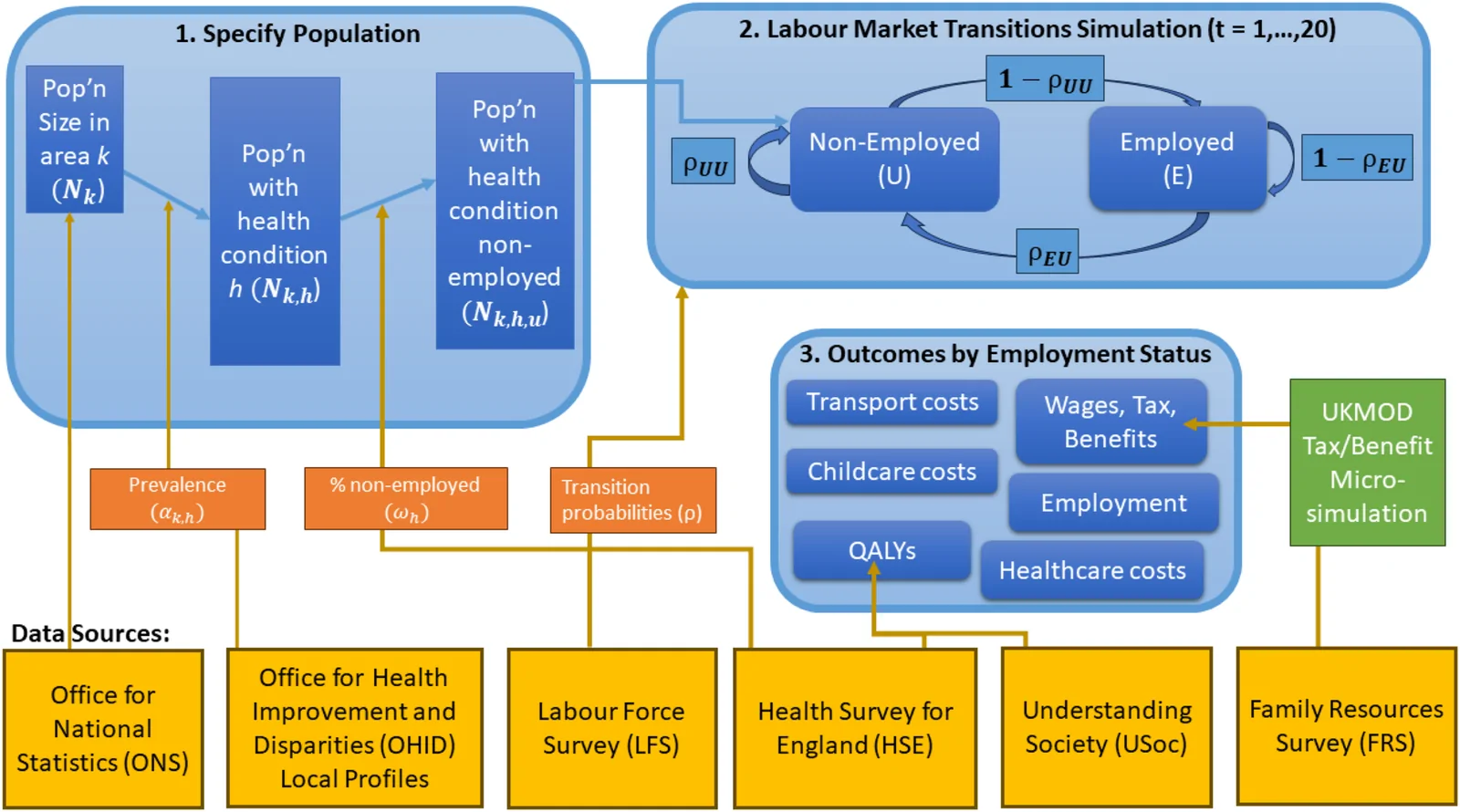
Schematic diagram for the Individual Placement Support Cost-Benefit Model including population specification, labour market transitions, outcomes and their valuation.

Health-related worklessness presents a significant economic challenge across OECD countries, with employment rates among people with chronic health conditions typically 20-30% lower than those without such conditions. [5,6] Employment rates for individuals with common mental health conditions are about 20% lower, while their likelihood of receiving out-of-work benefits is approximately 45% higher. [7,8] Given that mental illness affects around 20% of the working-age population across the OECD [7,8] this employment gap represents a substantial loss of productivity.

Addressing the relationship between health and work has important individual, economic and social implications. [9] These challenges cost the UK nearly £200 billion each year through lost productivity, higher NHS treatment costs, increased benefit spending and lower tax revenues. [10] Unemployment is both a cause and consequence of poor health, [11] leading to worsened mental health, reduced quality of life, and increased poverty. In particular, those out of work are two to three times more likely to experience mental health problems than those employed. [6] In contrast, paid employment can improve mental health through social inclusion and connection, and is linked to reduced suicide risk, relapse, and better physical health. [[12], [13], [14], [15], [16]]

IPS is an evidence-based model of supported employment, and consistently outperforms alternative approaches in improving employment outcomes. [12,17,18] It follows a “place then train” approach, focusing on rapid placement into competitive employment tailored to individual preferences, with ongoing health and practical support. [19,20] IPS is typically delivered by employment specialists embedded within clinical teams, who work proactively with employers to find well-matched roles.

IPS was originally developed for people with severe mental illness but has since been expanded to include other health conditions and complex disadvantages. England has been at the forefront of this innovation, with large-scale randomised controlled trials (RCTs) contributing substantially to the international evidence base and helping to reshape UK employment policy. [9,21] Most recently, trials in England have focused on people with low-to-moderate mental health (MH) and musculoskeletal (MSK) conditions. [22]

Despite growing evidence of IPS effectiveness, evidence on its return on investment remains limited. [12] A recent systematic review found that 79% of 56 studies reported a positive economic case. [23] However, few evaluations use long-term modelling, disaggregate results by population subgroups, or conduct sensitivity analyses around intervention costs or effectiveness. These limitations make it challenging for policymakers and commissioners to assess the economic case for IPS.

This study addresses these gaps by developing the IPS cost-benefit and outcomes model (IPS-MOD), [24] a new open-source modelling tool. We evaluate the long-term health and economic impacts of IPS for people in England with: (i) low-to-moderate MH conditions, (ii) MSK conditions, and (iii) combined MH + MSK conditions. Specifically, we ask: (1) What are the health and economic impacts of IPS for these populations over five years? and (2) How do these impacts vary under different assumptions about service cost and effectiveness?

## 2 Methods

### 2.1 Model overview

IPS-MOD is an Excel-based tool which models three priority health condition subgroups of non-employed individuals with MH, MSK and MSK + MH. The model adheres to the HM Treasury Green Book guidelines for policy and program appraisals. [25] This section gives an overview of the IPS-MOD framework and its data sources, with more detail on the methods used available in the Supplementary appendix part A.

IPS-MOD compares outcomes of a cohort of individuals in a scenario where they received the IPS intervention to a control scenario in which they received standard UK employment support (principally Jobcentre Plus provision). Individuals are defined as employed or non-employed, and each state is associated with a profile of economic and health economic outcomes. Differences in outcomes between the intervention and control arms occur due to (i) differing transition probabilities between states and (ii) different average QALYs. The model first defines the number of non-employed individuals in the health condition subgroup. It then simulates employment transitions as a Markov process for each health condition over five years in quarterly time steps (20 cycles; Supplementary appendix Figure S6). Outcomes include QALYs, healthcare costs, employment, net earnings, income taxes, social security benefit payments, and employment-related childcare and transport costs. Default discount rates applied are 3.5% for financial costs and benefits and 1.5% for health benefits. Discounted costs and benefits produce benefit-cost ratios (BCRs) measuring the IPS service's return on investment (ROI).

### 2.2 Health-led employment trial

The Department for Work and Pensions' (DWP's) Health-Led Employment Trial (HLET) [26] was designed to test the effectiveness of extending Individual Placement and Support (IPS) to people with long-term health conditions beyond severe mental illness. The large-scale trial evaluated IPS for individuals with low-to-moderate mental health (MH), musculoskeletal (MSK), or combined MH and MSK conditions, recruited from two regions in England. Participants were randomly assigned to receive IPS or usual support, with outcomes assessed using survey and administrative data to examine impacts on employment, health, and wellbeing. [27]

### 2.3 Population size

We use mid-year population estimates from the Office for National Statistics (ONS) to determine the working-age population by local authority. These are combined with local authority-level estimates of MSK, MH, and MSK + MH condition prevalence. [28] Using the 2019 Health Survey for England (HSE), [29] we estimate the number of non-employed individuals in these health-condition subgroups. These estimates form the IPS-MOD default population size for our labour market simulation.

### 2.4 Labour market transitions

Modelling the baseline scenario involves estimating transition probabilities between employment states (employed and non-employed) using 27 five-wave longitudinal datasets from the Labour Force Survey (LFS) spanning Q2 2012 to Q4 2018 (this period includes health condition information). We calculate transition probabilities for individuals who start non-employed in wave 1. We do this for six subgroups categorised by health condition and binary disability status, with disability defined as being in receipt of disability-specific social security benefits. The LFS tracks individuals for five quarters with 13-week intervals, allowing us to observe labour market transitions over one year. We construct a detailed one-year decision tree model (Supplementary appendix Figure S5), with transitions after the first year modelled as a Markov process based on pooled LFS transition probabilities. We calibrated the LFS-based transition probabilities by applying adjustment factors so that the model reproduces the year-1 employment outcomes observed in the HLET for both the control and treatment arms (Supplementary appendix Table S7 to S8).

### 2.5 QALYs

Health outcomes are measured using QALYs, a standard metric in health economics combining both the quality and length of life. One QALY represents one year of life in perfect health, with lower values reflecting poorer health states. IPS impacts QALYs in two ways: the indirect impact of improved health through increased employment, and the direct effect on health utility for both employed and non-employed individuals from participation in IPS. The QALYs are estimated from EQ-5D by health condition for the non-employed using 2019 HSE data. [30] For the employed population, we conducted regression analyses using Understanding Society data (USoc) [31] to model EQ-5D as a function of employment status and other controls (Supplementary appendix p14 to p16). The regression coefficient for employment status is used to adjust the EQ-5D score of the non-employed population to represent the EQ-5D improvement gained by moving into work in IPS-MOD (Supplementary appendix Table S3). To convert health outcomes into financial terms, each QALY is assigned a value of £70,000 (2020/21 prices), in line with the Green Book [25] recommendations and adjusted for inflation to £79,467.99 (2024 prices).

### 2.6 Taxes, benefits, and earnings

The UKMOD tax-benefit microsimulation model version B1.09, [32] is used to calculate earnings, tax payments, and social security benefit receipts based on the nationally representative Family Resources Survey. These outcomes are modelled by health condition and employment status subgroups, with the employed restricted to having been in work for less than one year. This produces a profile of tax, benefit, and income associated with each health condition and employment status subgroup in the model (Supplementary appendix Figure S4).

### 2.7 Other financial costs and benefits

Other financial costs and benefits related to an individual's transition from non-employment to employment include reductions in healthcare costs and increases in childcare and transport costs. These figures are sourced from the DWP Social Cost Benefit Analysis Framework [33] and adjusted for inflation.

### 2.8 Modelling years 2-5

While the trial evidence can only calibrate the model for Year 1, IPS-MOD extends over five years to capture persisting benefits. Due to a lack of data for calibration beyond Year 1, and the absence of published evidence on the persistence of IPS effects, IPS-MOD includes several user-selectable scenarios, these are presented in Supplementary appendix Figure S7. The default scenario assumes treatment effects diminish to 25% of their Year 1 value by Year 5, this reflects qualitative evidence that IPS aims not only to support job entry but also to build longer-term skills, motivation and resilience, [34] and offers continued support if difficulties arise in work. Direct treatment effects on health utility are assumed to be permanent. Supplementary appendix Table S19 shows the outcomes of the different year 2-5 scenarios.

### 2.9 Cost-benefit analysis

Having modelled labour market transitions over a five-year time horizon, we obtain monetary outcomes by associating each health condition and employment status at a given time with average earnings, taxes, benefits, QALYs, healthcare costs, transport costs and childcare costs per person. These outcomes are then multiplied by the number of individuals in each state and aggregated over the five years of the model. Government gains, individual gains and a societal perspective BCR is calculated in IPS-MOD (Supplementary appendix Table S11). The interpretation of the BCR is the amount received in societal benefits per £1 spent on IPS, e.g. A BCR of 1.10 indicates that for every £1 spent on IPS, society receives £1.10 in benefits, and therefore the intervention delivers a positive return on investment.

### 2.10 Scenarios

IPS-MOD models the economic impact of IPS services for individuals with three different health conditions: MSK, MH, and MSK + MH. We focus here on the MSK population, with IPS-MOD outcomes for MH and MSK + MH provided in Table 5 and Supplementary appendix Tables S13 to S16.

Table 1 illustrates the base-case scenario, where we assume an average sized local authority with a working-age population of 180,000 individuals. The estimated number of non-employed individuals with each health condition is 14,813 for MH, 12,498 for MSK, and 4956 for MH + MSK. Of these, we assume 30% voluntarily participate in IPS, resulting in a service population of 4444 for MH, 3749 for MSK, and 1487 for MH + MSK. This is a modelling assumption rather than observed uptake and can be changed by users. As it sets the cohort size in both arms, it affects population totals, such as aggregate QALY gains and exchequer savings, but not per-person outcomes or the BCR.

**Table 1:** IPS-MOD default parameters for the base-case scenario to estimate cost-benefit of implementing IPS for three specified health conditions in a typical sized local authority.

Health Condition	Low to moderate mental health conditions	Low to moderate musculoskeletal conditions	Low to moderate mental health conditions AND low to moderate musculoskeletal conditions
**Eligible population**			
Working age population (16 - 64)	180,000	180,000	180,000
Population with specified health condition	30,673^a^	31,542^a^	7648^a^
Non employed population with specified health condition	14,813^b^	12,498^b^	4956^b^
% Of non-employed population with specified health condition that is estimated would voluntarily join the IPS service	30%	30%	30%
**Population participating in IPS service.**			
Non employed population with specified health condition that is estimated would voluntarily join the IPS service	4444	3749	1487
**Population claiming sickness related benefits**^c^			
% Of participants claiming sickness related benefits	11%	11%	11%
Number of participants claiming sickness related benefits	489	433	172
**Service Cost & Service Effectiveness**^c^			
Service cost (per person)	£2326.00	£2326.00	£2326.00
% Of participants receiving IPS service that worked any point in the first 12 months	57%	28%	28%
% Of participants **not** on IPS service that worked any point in the first 12 months	53%	24%	23%
Valuation^d^			
Monetary Value of a QALY	£79,467.99	£79,467.99	£79,467.99
Discount rate (financial costs/benefits)	3.5%	3.5%	3.5%
Discount rate (health benefits)	1.5%	1.5%	1.5%

a Office for Health Improvement and Disparities (OHID) Local Profiles.

b Health Survey for England (HSE).

c Health Led Employment Trial (HLET).

d HM Treasury Green Book (2022).

The service cost per participant is set at £2326.00. Effectiveness is measured by the percentage of participants who gain employment within the first 12 months: 57% for MH, 28% for MSK, and 28% for MH + MSK. For comparison, in the absence of IPS, these employment rates are 53%, 24%, and 23%, respectively.

These base-case values provide a benchmark for cost-benefit analysis, with flexibility to adjust parameters and assess alternative scenarios, such as service cost, effectiveness, and participation rates.

### 2.11 Sensitivity analysis

To assess the robustness of the cost-benefit analysis (CBA), we conducted two-way sensitivity analyses, varying IPS service effectiveness (the percentage of participants who gained employment within the first 12 months) and service cost per participant. These analyses explore how changes in these parameters impact the BCR. We examined scenarios where IPS effectiveness ranged from 18% to 45%, alongside service costs from £1500 to £3300 per participant.

## 3 Results

We present detailed results for MSK conditions over a five-year period in terms of employment rates, average weeks of paid work, health utility changes, exchequer gains and societal perspective benefit-cost ratios. Outcomes beyond Year 1 are model projections based on the assumptions set out in the Methods. The equivalent detailed results for MH and for MSK + MH combined populations are shown in Table 5 and Supplementary Appendix Tables S13 to S19.

### 3.1 Base case results for population of people living with musculoskeletal conditions

Table 2 first shows employment effects. An eligible working-age population of 180,000 has been inputted, generating a default sample size of 3749 participants. At baseline (Year 0), both groups exhibited 0% employment. By the end of Year 1, the employment rate for those receiving IPS services increased to 21.55%, compared to 16.73% for those without IPS services. This represents a 4.82% absolute increase, corresponding to a 28.83% relative gain. By the end of Year 5, the employment rate for the IPS group reached 54.29%, higher than the 51.28% observed in the non-IPS group, reflecting a 3.01% absolute gain and a 5.87% relative gain. By the end of Year 1, individuals in the IPS group had worked an average of 7.74 weeks, 1.47 weeks more than the 6.27 weeks averaged by the non-IPS group. By the end of Year 5, those receiving IPS services worked an average of 104.11 weeks, an 8.65-week increase over the 95.46 weeks for the non-IPS group.

**Table 2:** IPS-MOD Results for a typical local authority for Musculoskeletal Conditions (3749 Participants). Effects on employment, health, exchequer, and individual income.

	Year 0	By the end of year 1	By the end of year 5
**Probability of being employed (%)**			
Without IPS service	0.00%	16.73%	51.28%
With IPS service	0.00%	21.55%	54.29%
Gains from IPS (absolute change)	0.00%	4.82%	3.01%
Gains from IPS (% change)	0.00%	28.83%	5.87%
**Average weeks in which paid work took place per person**			
Without IPS service	0	6.27	95.46
With IPS service	0	7.74	104.11
Gains from IPS	0	1.47	8.65
**Health utility change**			
QALY gains to those receiving IPS service (per person average)		0.028	0.137
Value of QALY gains to those receiving IPS service (£) (per person average)		£2200.63	£10,881.11
Value of QALY gains to those receiving IPS service (Million £) (population total)		£8.251M	£40.796M
**Costs and benefits to government (population total)**			
Savings in National Health Service (NHS) spending		£84,669.77	£467,105.09
Savings in social security payments		£686,353.84	£3,786,467.96
Increases in direct tax (income tax + council tax)		£211,281.53	£1,165,595.19
Increases in employee/self-employed national insurance.		£79,728.56	£439,845.47
Increases in employer national insurance.		£139,063.79	£767,185.29
Total Benefits (Million £)		£1.201M	£6.626M
**Individual gains (per person average)**			
Cumulative gains (£) to those on IPS service		£2352.41	£11,718.45
**Societal perspective benefit cost ratio**			
Every £1 spent on IPS service returns.		£1.13	£5.71

Health utility, measured in QALYs, improved for the IPS group, with average gains of 0.028 QALYs by the end of Year 1 and 0.137 QALYs by the end of Year 5. The corresponding monetary value of these QALY gains was £2200.63 per person by the end of Year 1 and £10,881.11 per person by the end of Year 5. Aggregated across the population, these values translated to £8.251 million and £40.796 million by the end of Years 1 and 5, respectively.

Table 2 also presents financial effects for government and for individuals. Exchequer gains, encompassing savings and increased revenues, indicated substantial financial benefits. By the end of Year 1, savings in NHS spending amounted to £84,669.77, and by the end of Year 5, this figure rose to £467,105.09. Savings in social security payments were £686,353.84 by the end of Year 1 and £3,786,467.96 by the end of Year 5. Increases in direct tax, employee/self-employed national insurance and employer national insurance contributed to further gains. The total benefits amounted to £1.201 million by the end of Year 1 and £6.626 million by the end of Year 5. Individual gains for those receiving IPS services were also noteworthy. By the end of Year 1, the cumulative gains per person were £2352.41, increasing to £11,718.45 by the end of Year 5.

Finally, the societal perspective benefit-cost ratio showed that every £1 spent on IPS services returned £1.13 by the end of Year 1 and £5.71 by the end of Year 5. Table 3 presents a breakdown of the components of the societal perspective benefit cost ratio as well as the effect IPS has on taxes and social security payments on an individual level. In summary, the IPS service improved employment outcomes, increased weeks of paid work, enhanced health utility, and yielded significant economic benefits from both individual and societal perspectives over the five-year period.

**Table 3:** IPS-MOD Results for a typical local authority for Musculoskeletal Conditions. Breakdown of benefits and costs by years and societal perspective benefit cost ratio (BCR).

	Year 1	By the end of year 2	By the end of year 3	By the end of year 4	By the end of year 5
**Costs (individual level)**					
**Total cost of IPS**	**£2326.00**	**£2326.00**	**£2326.00**	**£2326.00**	**£2326.00**
**Benefits (individual level)**					
A) Savings in National Health Service (NHS) spending	£22.58	£56.89	£87.17	£109.90	£124.59
B) Savings in social security payments	£183.06	£461.17	£706.65	£890.88	£1009.92
C) Increase to wages	£435.74	£1097.71	£1682.01	£2120.52	£2403.88
D) Value of gains to health utility	£2200.63	£4423.01	£6618.93	£8773.32	£10,881.11
E) Change in childcare	-£5.94	-£14.95	-£22.91	-£28.88	-£32.74
F) Change in transport	-£17.34	-£43.69	-£66.94	-£84.39	-£95.67
G) Increases in direct tax (income tax + council tax)	£56.35	£141.96	£217.53	£274.24	£310.89
H) Increases in employee/self-employed national insurance	£21.27	£53.57	£82.09	£103.49	£117.32
I) Increases in employer national insurance	£37.09	£93.44	£143.18	£180.50	£204.62
**J) Societal perspective total benefits (a + c + d + e + f)**	**£2635.67**	**£5518.97**	**£8298.26**	**£10,890.46**	**£13,281.17**
**Net benefit**	**£309.67**	**£3192.97**	**£5972.26**	**£8564.46**	**£10,955.17**
**Benefit cost ratio (BCR) (J divided by Total costs)**	**£1.13**	**£2.37**	**£3.57**	**£4.68**	**£5.71**

### 3.2 Sensitivity analysis for people living with musculoskeletal conditions

Table 4 shows the outcomes of the sensitivity analysis conducted on IPS-MOD for the MSK population. It presents the BCR under different scenarios of service cost and effectiveness. Negative ROI is indicated by red results; for instance, a BCR of 0.70 means that society receives £0.70 in benefits for every £1 spent on IPS, indicating a negative return on investment. Borderline values are shown in orange, while positive return outcomes are highlighted in green. The analysis demonstrates that economic outcomes are sensitive to both reductions in effectiveness and increases in service cost, with some scenarios resulting in negative ROI in the short term. Results show IPS delivers a positive return on investment in most modelled scenarios by the end of Year 1, and by the end of Year 5, all modelled scenarios result in a positive return on investment. For context, an employment entry target of approximately 40% is now typical for IPS programmes targeting these population groups in the UK. Further sensitivity analyses are presented in Supplementary appendix Table S19 and Fig. S9, including changes in the BCR based on different valuations of a QALY, as well as the BCR by the end of Year 5 under alternative assumptions about the duration of effect.

**Table 4:**
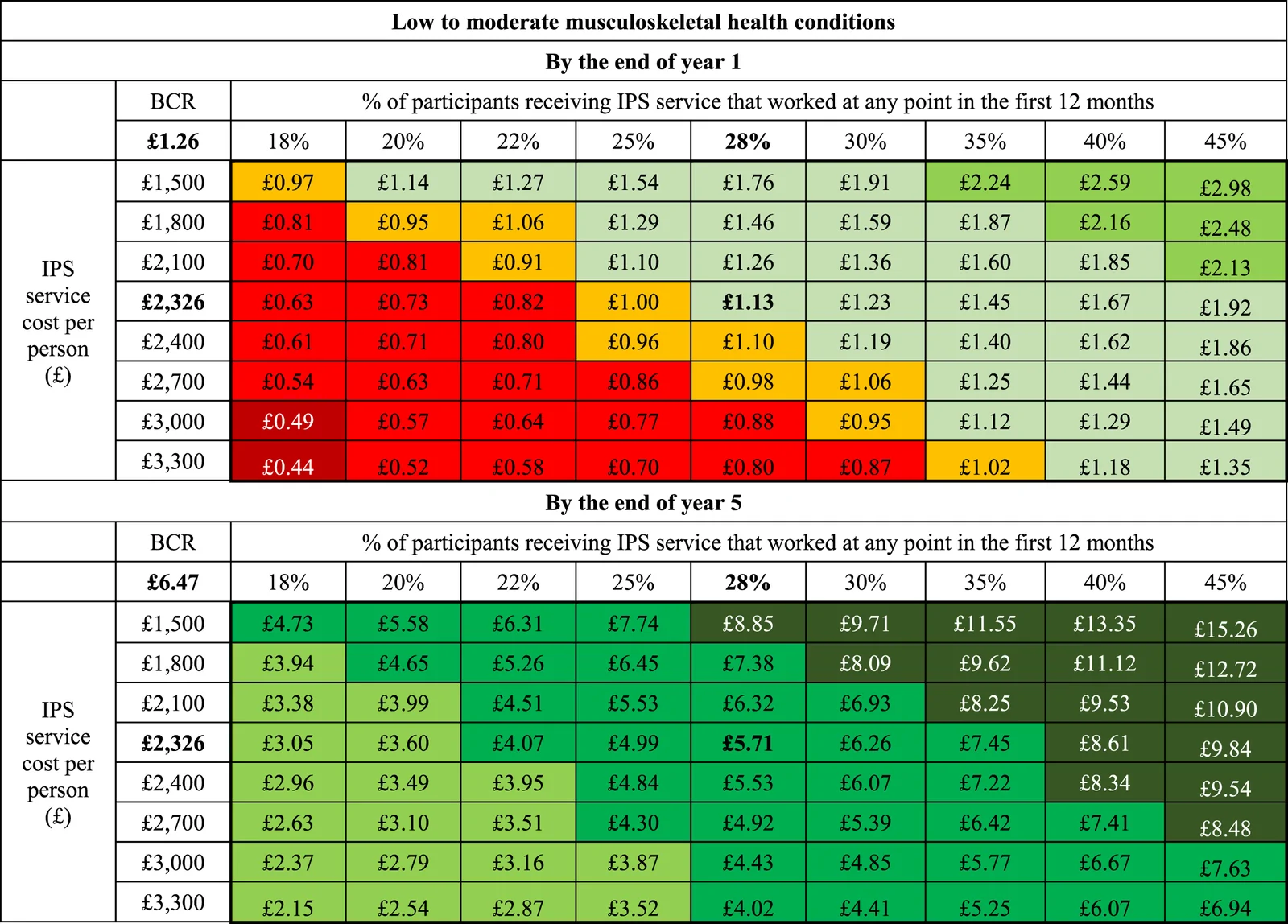
Example of Using IPS-MOD Tool to assess sensitivity of benefit-cost ratio to the IPS service cost per person.

### 3.3 Results for MH and MSK + MH groups

Table 5 presents the year 5 results for the MH and MSK + MH groups alongside the MSK group, with detailed results in Supplementary appendix Tables S13 to S16. The central estimate results are of a similar order of magnitude to those for the MSK group. For example, the one-year BCR is £1.22 for the MH group and £0.77 for the MSK + MH group. Over five years, the BCRs are £5.67 and £4.13, respectively. The MSK + MH group receives slightly less incremental benefit per person, mainly reflecting lower baseline employment transition rates for this group, based on LFS data and calibrated using results from the HLET. [26] We apply the same relative effect to the transition probabilities across health condition groups, resulting in a smaller absolute effect for the MSK + MH group due to their lower baseline transition rates. Sensitivity analyses on IPS cost per person and intervention effectiveness show a similar pattern to that observed for the MSK group (Supplementary appendix Tables S17 and S18). Further sensitivity analyses show consistent findings, including the BCR by the end of Year 5 under alternative assumptions about the duration of effect (Supplementary appendix Table S19) and changes in the BCR based on different valuations of a QALY (Supplementary appendix Figures S10 and S11).

**Table 5:** IPS-MOD Results for a typical local authority for Musculoskeletal, Mental Health, and Mental Health and Musculoskeletal Conditions. Differences between IPS and control arms in employment, health, social security payments, and benefit-cost ratio by the end of year 5.

By the end of year 5	Low to moderate musculoskeletal conditions (3749 Participants)	Low to moderate mental health conditions (4444 Participants)	Low to moderate mental health conditions AND low to moderate musculoskeletal conditions (1487 Participants)
Probability of being employed (%)			
Gains from IPS (absolute change)	3.01%	2.16%	4.36%
**Average weeks in which paid work took place per person**			
Gains from IPS	8.65	4.63	10.24
**Health utility change**			
QALY gains to those receiving IPS service (per person average)	0.137	0.150	0.0939
**Savings in social security payments (Population total)**			
Savings in social security payments	£3,786,467.96	£2,299,752.06	£1,757,488.85
**Societal perspective benefit cost ratio**			
Every £1 spent on IPS service returns.	£5.71	£5.67	£4.13

## 4 Discussion

This study introduces IPS-MOD, a new open-source cost-benefit analysis tool for IPS services for workless individuals with MH and/or MSK in England. Our case study demonstrates that implementing IPS for these individuals leads to improvements in employment rates, health outcomes, and economic benefits, with positive BCRs observed across all health condition groups by Year 5. Health utility gains were the primary driver of returns, followed by increases in wages. Although monetised QALY gains make up a large proportion of the estimated benefits, our sensitivity analyses suggest a positive return by Year 5 would be maintained for QALY values above approximately £10,000 (Supplementary appendix Figures S9 to S11), well below the Green Book valuation used in our base case.

Given the pace of IPS innovation, there are inevitably new studies appearing on a regular basis. The most relevant UK studies are the IPS-Alcohol and Drugs (IPS-AD) trial [35] and the HLET. [26] When configured to match the HLET population and costs, IPS-MOD closely reproduced the trial's year 1 employment outcomes and benefit-cost ratio (Supplementary appendix Table S20). Extending these estimates from one to five years, our results align with the existing evidence base that IPS services deliver substantial economic returns to the individual, society and government at realistic service costs and performance levels. The IPS-MOD tool extends the literature by offering more detailed, disaggregated, and flexible analyses for use in research, policy, and practice.

IPS-MOD has several strengths. It is evidence-based, combining findings from RCTs, survey datasets, the Green Book and the DWPs Social Cost-Benefit Analysis framework. It breaks down financial costs and benefits including healthcare service savings, social security savings, increased wages, health utility gains childcare and transport costs, and income tax receipts. Benefits are modelled over a five year horizon, which offers a more complete long-term view, in contrast to previous studies that often focus on a one-year window [26] Finally, its sensitivity analysis functions allow users to vary service performance and costs, enabling an understanding of its impacts on the benefit-cost ratio. IPS-MOD is open source, so it can be adapted to other settings and updated as new evidence becomes available.

IPS-MOD has several limitations, primarily due to underlying data constraints. While probabilistic sensitivity analysis would ideally be used to generate confidence intervals for the model's outputs, this was not possible due to the absence of reported distributions for key input parameters in the HLET data. [26] Instead, our structured two-way sensitivity analyses provide insight into how variations in key assumptions affect IPS-MOD's estimated cost-benefit outcomes. Secondly, uncertainty remains around longer-term effects beyond year one, because the published HLET evidence, against which IPS-MOD has been calibrated, provides only a 12 month impact evaluation. [22] We address this by providing varying user-selectable scenarios for longer-term effectiveness beyond year one whilst defaulting to a more conservative scenario of gradually diminishing intervention effects in the base case, informed by prior research. [21,23]

A third limitation is that IPS-MOD assumes that performance outcomes observed in the two HLET regions (South Yorkshire and West Midlands) are representative of expected performance nationally. This applies both to an established IPS service and to a newly implemented service, such as those in the trial. Given that performance tends to improve as IPS services establish, [17] IPS-MOD might therefore underestimate potential savings in established IPS services, although our expectation is that IPS-MOD will mostly be used to test and/or evaluate newer services. The inclusion of user customisable performance values helps to partially mitigate this limitation.

A fourth limitation is the assumption that the relative treatment effect on labour market outcomes observed in the HLET (Supplementary appendix Table S7) applies equally across all modelled health conditions, MSK, MH, and MSK + MH, as well as across disability statuses, including those claiming and not claiming sickness benefits.

Finally, as a cohort Markov model with two states, employed and non-employed, IPS-MOD does not distinguish between types of employment. The health benefits of work are likely to depend on its quality, security and sustainability, so modelled health gains may differ where jobs gained are insecure or of poor quality.

Future research can help address these limitations. First, studies should report key model parameters more consistently and in greater detail. For example, reporting differentiated sub-group volumes and outcomes as well as reporting confidence intervals and probabilistic distributions would enhance modelling accuracy. Second, trial designs should incorporate longer-term follow-ups to better understand long-term impacts. Third, additional studies in different regions of England and internationally would allow for the model to be calibrated for different contexts and improve its generalisability.

Our study has several implications for policy and practice. The evidence base is clear that IPS is a highly effective model of employment support for people with a range of health conditions, disabilities, and complex disadvantages. [12,17,21] However, demonstrated effectiveness is only one factor influencing commissioning decisions. IPS services often have a higher per-person cost than standard business-as-usual provision, so decision-makers must weigh these costs against expected returns. Commissioners also need to understand the distribution of economic impacts across stakeholders. This information can support decision-making in constrained budgets, align stakeholders where benefits are shared, strengthen the case for investment, and assess how costs and benefits fall on individuals, local authorities, health systems, and central government. IPS-MOD provides these functions for policymakers in England, using the best available evidence, and shows that IPS is likely to deliver significant and growing financial savings across multiple stakeholders. As the £1 billion Connect to Work programme scales up IPS for jobseekers with low-to-moderate MH and/or MSK conditions across England and Wales, IPS-MOD offers a valuable resource to support local and regional partners in commissioning, delivery, and evaluation.

The methodological approach taken in IPS-MOD is generalisable to a wide range of geographical contexts and health conditions. Detailed description of the code and technical documentation alongside the Excel tool facilitates further extension and adaptation. The IPS-MOD framework developed here could be generalised to any country with a national health survey (like HSE) and a longitudinal labour survey (like LFS). The specific findings from our case-study are not generalisable to other settings, health conditions or countries. The benefit-cost ratios fundamentally depend on the baseline probabilities of entering and remaining in employment, and the difference that IPS makes to those probabilities for the specified conditions in the context of the UK labour market.

### 4.1 Conclusions

IPS-MOD is a flexible, open-source, evidence-based economic evaluation tool designed to inform IPS service planning for non-working individuals with MH and/or MSK conditions. It enables users to model cost-benefit outcomes under a range of scenarios regarding programme size, cost per participant, performance level, and long-term effects assumptions. IPS-MOD can support central and local stakeholders across England to make evidence-informed decisions on commissioning and expansion of IPS services. Similar approaches could also be adapted in other contexts to strengthen evaluation and advance the development of effective, cost-beneficial employment support programmes.

## Ethical statement

Ethical approval was not required for this study. IPS-MOD is a cost-benefit modelling tool that uses only previously published trial results and pre-existing, anonymised secondary data sources, including the Labour Force Survey, Health Survey for England, Understanding Society and the Family Resources Survey. No primary data were collected from human participants or animals.

## Funding

This study was funded by the National Institute for Health and Care Research (NIHR) (grant number 202996). The views expressed are those of the authors and not necessarily those of the NIHR or the Department of Health and Social Care.

## Declaration of competing interest

The authors declare that they have no known competing financial interests or personal relationships that could have appeared to influence the work reported in this paper.
