# Supplementary material for: Estimating the health and economic impacts of Individual Placement and Support (IPS) for people with mental and physical health conditions: A cost-benefit modelling study
Source: Public Health Pract (Oxf). 2026 Oct 6;12:100867. doi: 10.1016/j.puhip.2026.100867 (PMC13678004; doi:10.1016/j.puhip.2026.100867)
Supplement: Supplementary file 1 [file mmc1.docx]

**Estimating the Health and Economic Impacts of Individual Placement and Support (IPS) for People with Mental and Physical Health Conditions: A Cost-Benefit Modelling Study**

**Supplementary Appendix**

Contents

[Part A: Methods	5](#_Toc241238430)

[1.	Introduction	5](#_Toc241238431)

[2. Data	5](#_Toc241238432)

[2.1 Population data	5](#_Toc241238433)

[2.2 Public health profiles for England	6](#_Toc241238434)

[2.3 Family Resources Survey	6](#_Toc241238435)

[2.4 Health Survey for England	7](#_Toc241238436)

[2.5 Understanding Society: The UK Household Longitudinal Study	7](#_Toc241238437)

[2.6 Labour Force Survey	8](#_Toc241238438)

[2.7 Other Data Sources	10](#_Toc241238439)

[2.7.1 The DWP Social Cost-Benefit Analysis framework	10](#_Toc241238440)

[2.7.2 The Work and Health Unit Health Led Employment Trial	10](#_Toc241238441)

[2.7.3 The Green Book	10](#_Toc241238442)

[3. Modelling the Baseline	10](#_Toc241238443)

[3.1 Overview	11](#_Toc241238444)

[3.2 Defining the Eligible Population	13](#_Toc241238445)

[3.2.1 Calculating the population with a health condition	13](#_Toc241238446)

[3.2.2 Calculating the population with a health condition and OOW	13](#_Toc241238447)

[3.3 Modelling Health Utilities	14](#_Toc241238448)

[3.3.1 The EQ-5D	14](#_Toc241238449)

[3.3.2 Calculating EQ-5D utilities	14](#_Toc241238450)

[3.3.3 Estimating the health utility gain from transitioning into employment	15](#_Toc241238451)

[3.4 Modelling Tax and Benefits	16](#_Toc241238452)

[3.4.1 UKMOD	16](#_Toc241238453)

[3.4.2 Modelling approach	16](#_Toc241238454)

[3.4.3 Modelled Parameters for Income, Tax, and Benefits	18](#_Toc241238455)

[3.5 Other Financial Costs and Benefits	19](#_Toc241238456)

[3.5.1 Transport costs	19](#_Toc241238457)

[3.5.2 Childcare costs	19](#_Toc241238458)

[3.5.3 NHS Spending	20](#_Toc241238459)

[3.6 Modelling labour market transitions	20](#_Toc241238460)

[3.6.1 Year One	21](#_Toc241238461)

[3.6.2 Years Two to Five	23](#_Toc241238462)

[4. Modelling the IPS intervention and control scenarios	25](#_Toc241238463)

[4.1 Adjusting transition probabilities for treatment effects	25](#_Toc241238464)

[4.1.1 Year One	25](#_Toc241238465)

[4.1.2 Years Two to Five	28](#_Toc241238466)

[4.2 Adjusting health utilities for treatment effects	29](#_Toc241238467)

[4.3 Allowing for differential performance	30](#_Toc241238468)

[4.4 Cost-Benefit Analysis	32](#_Toc241238469)

[4.5 IPS-MOD Inputs and Outputs	33](#_Toc241238470)

[Part B: Supplementary Results	34](#_Toc241238471)

[5. Introduction	34](#_Toc241238472)

[5.1 Base case Results for Population of People Living with Mental Health Conditions	34](#_Toc241238473)

[5.2 Base case Results for Population of People Living with Mental Health and Musculoskeletal Conditions	35](#_Toc241238474)

[5.3 Sensitivity Analysis	40](#_Toc241238475)

[5.4 Validating IPS-MOD against Health-Led Employment Trial Outcomes	47](#_Toc241238476)

Tables and Figures

[Table S1 Summary of sources for health condition prevalence data	6](#_Toc241238477)

[Figure S1: Structure of the Labour Force Survey quarterly and longitudinal datasets	8](#_Toc241238478)

[Figure S2: Schematic diagram for the Individual Placement Support Cost-Benefit Model including population specification, labour market transitions, outcomes and their valuation	12](#_Toc241238479)

[Figure S3: Calculating the eligible population to receive the IPS intervention	13](#_Toc241238480)

[Table S2 Non-employment rates by health condition in the 2018 Health Survey for England	14](#_Toc241238481)

[Table S3 Estimated EQ-5D utilities by health condition and employment status	16](#_Toc241238482)

[Figure S4: UKMOD summary outputs for average monthly earnings, benefits, and taxes by health condition and work status	18](#_Toc241238483)

[Table S4 Summary of other financial costs and benefits included in IPS-MOD	19](#_Toc241238484)

[Table S5 Summary of changing costs per week of moving from non-employment to full-time employment by health condition	20](#_Toc241238485)

[Figure S5: Example transition probability tree for year one of the labour market simulation	22](#_Toc241238486)

[Figure S6: Markov model structure	24](#_Toc241238487)

[Table S6 Average Weeks Worked per Quarter by Employment Status, Health Condition, and Benefit Status	24](#_Toc241238488)

[Table S7 Labour market outcomes used for model calibration	26](#_Toc241238489)

[Table S8 Transition probability calibration factors	27](#_Toc241238490)

[Table S9 Trial Demographic	27](#_Toc241238491)

[Figure S7: Different scenarios for the effect of IPS in years 2-5 post-receipt relative to the year 1 effects	29](#_Toc241238492)

[Table S10 IPS-MOD health utilities by health condition, employment, and arm	30](#_Toc241238493)

[Figure S8: Calibration factors for each employment rate - MSK & MH	31](#_Toc241238494)

[Table S11 Benefits and Costs, societal perspective benefit cost ratio, government gains and individual gains	32](#_Toc241238495)

[Table S12 IPS-MOD Inputs and Outputs	33](#_Toc241238496)

[Table S13: IPS-MOD Results for a typical local authority for Mental Health Conditions (4444 Participants). Effects on employment, health, exchequer, and individual income.	35](#_Toc241238497)

[Table S14: IPS-MOD Results for a typical local authority for Mental Health and Musculoskeletal Conditions (1487 Participants). Effects on employment, health, exchequer, and individual income.	37](#_Toc241238498)

[Table S15 IPS-MOD Results for a typical local authority for Mental Health Conditions. Breakdown of societal perspective benefit cost ratio (BCR) by items of benefit and by years.	38](#_Toc241238499)

[Table S16 IPS-MOD Results for a typical local authority for Mental Health and Musculoskeletal Conditions. Breakdown of societal perspective benefit cost ratio (BCR) by items of benefit and by years.	39](#_Toc241238500)

[Table S17 Example of Using IPS-MOD Tool to assess sensitivity of benefit-cost ratio to the IPS service cost per person - Low to moderate mental health conditions	41](#_Toc241238501)

[Table S18 Example of Using IPS-MOD Tool to assess sensitivity of benefit-cost ratio to the IPS service cost per person - Low to moderate mental health conditions AND low to moderate musculoskeletal conditions	42](#_Toc241238502)

[Table S19 BCR By the end of year 5 alternative scenarios on duration of effect	43](#_Toc241238503)

[Figure 9 Societal perspective Benefit Cost Ratio by Value of a QALY for Low to moderate musculoskeletal conditions	44](#_Toc241238504)

[Figure 10 Societal perspective Benefit Cost Ratio by Value of a QALY for Low to moderate mental health conditions	45](#_Toc241238505)

[Figure 11 Societal perspective Benefit Cost Ratio by Value of a QALY for Low to moderate mental health conditions AND low to moderate musculoskeletal conditions	46](#_Toc241238506)

[Table S20 Validating IPS-MOD against Health-Led Employment Trial Outcomes	48](#_Toc241238507)

# **Part A: Methods**

# 1. **Introduction**

This section describes the data sources, assumptions, and methodologies used to construct IPS-MOD, an economic model simulating the health and economic outcomes of individuals in England who are out of work (OOW) and have low-to-moderate mental (MH) and musculoskeletal (MSK) health conditions, or both (MSK+MH). The model estimates the impact of Individual Placement and Support (IPS) interventions on these outcomes to inform a cost-benefit analysis (CBA). IPS-MOD simulates labour market dynamics over five years, modelling quarterly transitions in and out of employment. It tracks employment rates, tax and benefit profiles, earnings, health-related quality of life (HRQoL) in quality-adjusted life years (QALYs), and associated costs such as healthcare, childcare, and transport. The model is calibrated using data from the Health-Led Employment Trials (HLTs), constructing IPS and control arms to estimate IPS effects on employment transitions and QALYs. These estimates are then extrapolated beyond the 12- and 22-month trial outcomes to a five-year horizon. The model also allows for scenario analyses, enabling users to input alternative IPS costs and performance metrics. The following sections detail the data sources (Section 2) and the labour market simulation methods (Section 3).

# **2. Data**

This section summarises the sources used for the data analysis and parameters used in constructing the model.

## **2.1 Population data**

The size of the working age population by local authority was taken from [Office for National Statistics](https://www.ons.gov.uk/employmentandlabourmarket/peopleinwork/employmentandemployeetypes/datasets/locallabourmarketindicatorsforcountieslocalandunitaryauthoritiesli01). The working age population is defined as those aged 16-64.

Populations are defined at the upper-tier local authority (UTLA) level, of which there are currently 152 in England. Throughout, we use the term “local authority” to refer to UTLAs. As the boundaries of some of these local authorities have changed, we process relevant data to fit a consistent set of local authorities. Some data sources are from periods with different local authority boundaries and must be adjusted to match the current boundaries. Changes to local authority boundaries which affect some of our data sources are:

- Bournemouth, Christchurch, and Poole. This unitary authority used to be two separate metropolitan districts (Bournemouth and Poole), and one non-metropolitan district (Christchurch). Where necessary, we construct the unitary authority by aggregating or taking the average across the three to produce a combined Bournemouth, Christchurch, and Poole figure.
- Dorset and Buckinghamshire were changed from counties to unitary authorities, and their area codes updated accordingly. In data where these areas are still classified as counties, we simply update the area codes to their unitary authority figures.
- Northamptonshire was split into North Northamptonshire and West Northamptonshire. For prevalence/proportions, we apply the overall figure for Northamptonshire to successor unitary authorities. For totals, we split the figure between the two in proportion to the populations of the two areas.

## **2.2 Public health profiles for England**

Prevalence data for MSK, MH, and MSK+MH are sourced from the [Office for Health Improvement and Disparities (OHID)](https://fingertips.phe.org.uk/) local profiles, processed in R/RStudio and extracted using the fingertipsR R package^2^. We obtain local authority level data on the prevalence of mental health conditions, musculoskeletal conditions, and both types of condition simultaneously.

| Fingertips Id | Current Use | Description | Source |
| --- | --- | --- | --- |
| 93377 | Prevalence of musculoskeletal conditions | ‘’Percentage reporting a long-term Musculoskeletal problem’’ | GP Patient Survey |
| 93495 | Prevalence of mental health conditions | ‘’Prevalence of Common Mental Health Disorders (% of population aged 16 and over)’’ | 2014 Adult Psychiatric Morbidity Survey (APMS) |
| 93372 | The prevalence of both musculoskeletal and mental health conditions simultaneously | “% with Long term MSK also reporting depression or anxiety.’’ 2016/17. | GP Patient Survey |

Table S1 Summary of sources for health condition prevalence data

The data on prevalence of musculoskeletal conditions is taken from Indicator 93377, “Percentage reporting a long-term Musculoskeletal problem”. MSK prevalence data are taken for the year 2022 and are representative of the population aged 16 or above. The data on prevalence of mental health conditions is taken from Indicator 93495, “Prevalence of Common Mental Health Disorders (% of population aged 16 & over)”. The most recent year of data available for this indicator is from 2017.

Our measure of the prevalence of both musculoskeletal and mental health conditions simultaneously is based on indicator 93372 – “Long term MSK also reporting depression or anxiety.” Data for this indicator are taken for the most recent available period, which is 2016/17. Unlike the prevalence figures for MH and MSK separately, the prevalence figures for this indicator relate to those aged 18 or above, rather than 16. This prevalence figure is the percentage of individuals who have long term musculoskeletal problems that also report anxiety or depression. To estimate the number of people in a local authority with both MSK and MH health conditions, we therefore first multiply the population size by the MSK prevalence, and then multiply that figure by the MSK+MH prevalence.

## **2.3 Family Resources Survey**

The Family Resources Survey (FRS)^3^ is an annual cross-sectional survey which collects detailed information about the incomes and assets of a representative sample of UK households, surveying around 20,000 individuals per year sampled across the fiscal year (April to March). The Family Resources Survey (FRS) is a continuous survey that was launched in 1992 to meet the information requirements of Department for Work and Pensions (DWP) analysts. The survey aims to: support the monitoring of the social security programme; support the costing and modelling of changes to national insurance contributions and social security benefits; and provide better information for the forecasting of benefit expenditure.

The FRS is the data on which the UKMOD tax-benefit micro-simulation tool is built, and is used in to derive tax, social security benefit, and net income profiles for subgroups defined by health condition and employment status (Section 3.4).

## **2.4 Health Survey for England**

The Health Survey for England (HSE) is an annual cross-sectional survey covering health and health-related behaviours. It also contains socio-economic information including age, sex, deprivation, and economic status. The HSE is a cross-sectional survey that monitors trends in health conditions and health behaviours of adults and children in England. The survey has run every year since 1991, except for 2020 due to the Covid-19 pandemic. The HSE is carried out by NHS digital and is run conjointly by the Joint Health Surveys Unit of NatCen Social Research and the Research Department of Epidemiology and Public Health at UCL. The data are collected from adults aged 16 and over, and children aged 0 to 15 through face-to-face in-home interviews and self-completion questionnaires. Approximately, 8,000 adults and 2,000 children participate in the survey every year.

To derive parameters for the model, the 2019^4^ HSE data was used. The HSE data are used to estimate the non-employment rates of individuals with MSK, MH, and MSK+MH health conditions (Section 3.2.2). These rates are combined with the local authority-level population data to calculate the number of non-employed individuals with each health condition to establish the size of the population which receives the IPS intervention.

## **2.5 Understanding Society: The UK Household Longitudinal Study**

The Understanding Society (USoc hereafter) dataset is a longitudinal survey of UK households which has run for 13 waves (as of November 2023). The data collected by the survey is wide in scope and includes health and well-being, income, education, labour market outcomes, and attitudes/beliefs. The USoc data is used to estimate the health utility gains from moving into work (Section 3.3). These data are used because the longitudinal nature of the data make it possible to follow individuals over time transitioning between employment states and changes in their self-reported health utility.

Econometric panel data techniques are used to estimate the health utility gain from moving into employment while controlling for confounding observable factors and unobserved time-invariant individual heterogeneity. These estimates are used to adjust the baseline non-employment health utilities estimated from the HSE data into the health utilities for those same individuals if they were to gain employment.

Figure S1: Structure of the Labour Force Survey quarterly and longitudinal datasets

## **2.6 Labour Force Survey**

The Labour Force Survey (LFS) is a long-running representative survey of all individuals in the UK that has taken place every quarter since 1992. It contains detailed demographic information about the respondents, and detailed data on individuals’ educational qualifications, employment status, occupation and industry of employment, spells of unemployment, hours worked, and earnings.

The LFS is released as quarterly cross-sectional datasets, but the survey design also incorporates a staggered longitudinal element. Each quarter, a new wave of respondents is interviewed and then followed up for four successive quarters for a total of five quarters. In addition to the quarterly cross-sectional data, longitudinal datasets are released for each cohort of longitudinal respondents. In any one quarter, the LFS survey sample contains five longitudinal cohorts, each at a different wave of participation in the survey. Figure S1 illustrates the structure of the LFS datasets.

The use of the LFS longitudinal data allows us to observe the same individuals at five distinct time points, each located 13 weeks from the previous, and so covering a time span of one year. We make use of the longitudinal data to calculate the probability of transitions between employment states for different population subgroups (Section 3.6).

Detailed descriptions of specific variables used in the Labour Force Survey (LFS):

**PERSID**

- Personal Identification Number
- **Details/Usage**: A unique identifier assigned to each respondent to track individual responses across different waves of the survey.

**LGWT**

- Longitudinal Weight
- **Details/Usage**: A weight applied to the responses to ensure the survey results are representative of the population over time.

**CONMPY1**

- Year Started Working with Current Employer
- **Details/Usage**: Applies to all respondents who are employees. Indicates the year (last two digits) when the respondent began working with their current employer.

**WEEK**

- Week of the Survey
- **Details/Usage**: The specific week in each wave which the respondent participated in the survey.

**INECAC05**

- Basic Economic Activity (ILO Definition)
- **Details/Usage**: Analyses employment status and economic activities.
- **Categories Include:**
- Employee
- Self-employed
- Government employment & training programmes.
- Unpaid family worker
- ILO unemployed
- Inactive (various reasons)

**CONMON**

- Month started current job
- **Details/Usage**: Indicates whether the individual was continuously employed throughout the previous month.

**AGE1**

- Age of Respondent at the time of their first survey participation.
- **Details/Usage**: Filtering to work age population.

**SCHM121**

- Work schemes
- **Details/Usage**: Are any participants on a work programme.

**GRSSWK**

- Gross Weekly Earnings
- **Details/Usage**: Weekly income before taxes and deductions.

**TTUSHR1**

- Total Usual Hours Worked Per Week
- **Details/Usage**: Average number of hours worked per week, used to analyse labour market participation. Total usual hours worked in main job (including overtime).

**WNEFT111**

- Whether Employed Full-Time or Part-Time.
- **Details/Usage**: Employment status indicator, distinguishing between full-time and part-time work.

**HEAL**

- What health problems does the respondent have
- **Details/Usage**: Self-reported health status, used to analyse health trends in relation to employment.

**OOBEN**

- Main reason those not in employment claiming out-of-work benefits
- **Details/Usage**: Unemployment, sickness, lone-parent, other

## **2.7 Other Data Sources**

### 2.7.1 The DWP Social Cost-Benefit Analysis framework

The cost and benefit estimates for changes in employment status consider healthcare savings, childcare expenses, and travel costs. These values are based on the Department for Work and Pensions (DWP) Social Cost Benefit Analysis Framework^5^, uprated for inflation since the original valuation.

### **2.7.2 The Work and Health Unit Health Led Employment Trial**

In a recent study, the Work and Health Unit (WHU) evaluated the effectiveness of Individual Placement and Support (IPS) for people with mild to moderate mental and/or physical health conditions^1^. We used data from the Health Led Employment Trial (HLET)^6^ to analyse how IPS impacted job transitions, the health improvements gained from participating in IPS, and the overall cost of delivering the program.

### **2.7.3 The Green Book**

The Green Book^7^ is guidance issued by HM Treasury on how to appraise policies, programmes, and projects. In IPS-MOD, the Green Book provides the default monetary value of a QALY, as well as default discount rates for financial and health cost/benefits.

# **3. Modelling the Baseline**

In this section we describe the methods used to construct the baseline model, beginning with an overview of the model structure and components in Section 3.1. There are six key components to parameterising and constructing the model:

1. Identifying the non-employed population with health conditions of interest by local area (Section 3.2).
2. Derive EQ-5D health utilities for in-work and out-of-work individuals by health condition (Section 3.3).
3. Construct income, tax, and benefit profiles for in-work and out-of-work individuals by health condition (Section 3.4).
4. Calculate other financial costs and benefits associated with employment and non-employment (Section 3.5).
5. Model labour market transitions for initially non-employed individuals with health conditions (Section 3.6).

We then move onto describing the modelling of labour market transitions under the IPS intervention in (Section 4).

## **3.1 Overview**

An overview of the model structure is summarised in Figure S2.

The first step in the model is to identify the number of individuals in the population of interest. The population is defined as those who are (i) of working age who are (ii) currently out of work and (iii) have a musculoskeletal health condition and/or a mental health condition. By specifying either a local authority or England as a whole, the user can obtain a pre-constructed estimate of the population size, or the user can override the model defaults and supply their own population size.

In the second step, the model takes this initial population and simulates the progress of this cohort through the labour market over a period of five years. Labour market progression is modelled as a Markov process by which at a given time point individuals are in one of two labour market states - employed or non-employed - and between time $t$ and $t+1$ there are probabilities of either remaining in the current state or moving to another one. The model operates in time steps of one quarter in length. At each time step, the model records the number of individuals employment (E) and in non-employment (U). The size of the cohort is assumed to remain constant over the time horizon and there are no absorbing states (i.e. no one dies or retires).

In the third step, key outcomes are associated with each labour market state and costs and benefits are aggregated to the total number of individuals in the state at that time. The key health outcome is quality adjusted life-years (QALYs) measured by the EQ-5D, which is valued according to the willingness to pay for a QALY of £70,000 in 2021 prices, in line with the Green Book^7^. For each health condition the model includes a profile of net wages, income tax and national insurance contributions paid, and benefits received by those who are employed and those who are non-employed. The model also includes other financial costs and benefits associated with employment, including transport costs, childcare costs, and National Health Service (NHS) spending.

Figure S2: Schematic diagram for the Individual Placement Support Cost-Benefit Model including population specification, labour market transitions, outcomes and their valuation

The impact of the IPS intervention in the model is captured by implementing a control arm (in which all individuals receive “business-as-usual” [BAU] support provided by the Department for Work and Pensions) and an intervention arm (in which all individuals receive IPS in addition to BAU) version of the process summarised above. There are two key parameters which differ between the control and intervention arms which drive the intervention effect of IPS in the model.

The first channel by which the IPS intervention arm deviates from the control arm is the employment status transition probabilities. In the intervention arm of the model, probabilities of non-employment are lower (both the probability of remaining non-employed for those who are out of work, and the probability of transitioning to non-employment for those in work). This means that at a given time point $t$, the intervention arm has a higher employment rate than the control arm. Outcomes not directly impacted by IPS will therefore differ between the two arms to the extent that they differ by employment status.

The second mechanism is through which IPS improves outcomes relative to the control arm is through health-related quality of life. IPS improves health-related quality of life indirectly and directly. The indirect channel is improvement in health by increasing the proportion of people in work, with health utilities (used to derive QALYs) being higher for the employed than the non-employed (Section 3.3). This indirect effect is captured through the impact of IPS on transition probabilities described above. The direct effect is an impact of the IPS intervention itself on health, through improved mental well-being, irrespective of whether or not the intervention leads to improved employment outcomes for the individual.

Cost-Benefit Analysis (CBA) is conducted by computing the BCR (benefit cost ratio) which measures the financial return to the IPS intervention per £1 spent on the intervention. CBA measures all costs and benefits, including health, in monetary terms. A BCR ratio larger than one delivers more in benefits than the intervention costs, and less than one means the intervention delivers less in benefits than it costs.

## **3.2 Defining the Eligible Population**

The eligible population to receive the intervention consists of those of working age who (i) have either a musculoskeletal condition and/or a mental health condition and (ii) are not employed. To construct the eligible population for a given local authority we obtain the total population of the local authority and adjust this figure for the prevalence of the health condition and the non-employment rate of individuals with that health condition. This calculation and the data sources used are summarised in Figure S3.

Figure S3: Calculating the eligible population to receive the IPS intervention

Note that the model allows the user to specify the number of individuals they expect to participate in an employment programme, which will override the defaults in the model. The purpose of the model defaults is to provide an illustrative population size for specific local authorities of individuals who are out of work with the specified health condition.

### **3.2.1 Calculating the population with a health condition**

We calculate the number of individuals, $N_{\left( k,h \right)}$, from local authority $k$ with a given health condition $h$ (where $h$ is either MSK, MH, or MSK+MH) by multiplying the local authority level prevalence of that health condition, $\alpha_{\left( k,h \right)}$, obtained from the OHID local authority profiles by the total number of working age individuals in the local authority obtained from ONS, $N_{k}$:

$$N_{k,h}=N_{k}*\alpha_{k,h} \left( 1 \right)$$

### **3.2.2 Calculating the population with a health condition and OOW**

We obtain non-employment rates by health condition from the Health Survey for England 2019 data. The HSE data does not contain local authority identifies, so we calculate these at the population level as the weighted mean of a dummy variable equal to one for an individual who is not in employment and zero otherwise. The non-employment rate for health condition $h$, $\omega_{h}$, is multiplied by the local authority population with the health condition, $N_{\left( k,h \right)}$, calculated from Equation 1:

$$N_{k,h,u}=N_{k,h}*\omega_{h}=N_{k}\left( \alpha_{k,h}*\omega_{h} \right) \left( 2 \right)$$

The $\omega_{h}$ are calculated from the Health Survey for England 2018 data and presented in the Table below. Note that the non-employment rates used in the model do not vary by local authority - an aggregate figure is calculated for England and applied to all local authority populations.

| Population | MSK | MH | MSK + MH |
| --- | --- | --- | --- |
| 21.36% | 39.62% | 48.29% | 64.80% |

*Table S2 Non-employment rates by health condition in the 2018 Health Survey for* England

## **3.3 Modelling Health Utilities**

We model health utilities separately by health condition and by employment status. Health utility is likely to vary by employment status due to psychological well-being effects of gaining employment. This is shown in an analysis using German data^8^ which showed a negative and significant impact of transitioning into non-employment on psychological health, and a positive and significant impact of transitioning out of non-employment. No significant impacts were found for physical health. In this application we econometrically model the relationship between employment status and EQ-5D utility score, without disaggregating between physical and psychological health or between into-work and out-of-work transitions.

### **3.3.1 The EQ-5D**

To measure individual health-related quality of life we use the EQ-5D to construct a health utility which can be used to calculate quality-adjusted life-years (QALYs). The EQ-5D scores responses to descriptions of health under five headings; mobility, self-care, usual activities, pain/discomfort, and anxiety/depression. There are two versions of the EQ-5D; the EQ-5D-3L and EQ-5D-5L, which differ in the number of responses which can be chosen from under each dimension. The EQ-5D-3L defines 243 possible health states compared to 3,125 possible health states in the EQ-5D-5L.

Responses to the EQ-5D questionnaire can be combined into a single health utility be applying an algorithm to the responses to produce a value with a maximum value of one (indicating perfect health) and including zero which is a state equivalent to death. Values less than zero are also possible, which are interpreted as states worse than death. Values for each health state are obtained by surveying a representative sample of the general population and using experimental methods to elicit values for health states defined by samples of possible combinations of responses to the five dimensions of the EQ-5D. Statistical models are then used to develop an algorithm for scoring the health utility for any of the 3,125 possible health states defined by responses to the five dimensions^8^.

### **3.3.2 Calculating EQ-5D utilities**

The model requires EQ-5D utilities for six unique health states: one each for the MSK, MH, and MSK+MH sub-populations, separately for out of work (OOW) and in-work (IW) individuals. We use data from the Health Survey for England (Section 2.4) 2019 dataset to obtain health utilities for OOW MSK, MH, and MSK+MH health condition groups.

While we can observe individuals in the HSE data who are IW cannot use their utilities as an estimate of the utility gain from the OOW transitioning into employment. This is because the individuals in the IW group will differ from the OOW group and as the HSE data are cross-sectional we cannot observe the same individuals moving in and out of employment to estimate the utility gains from entering employment. If the IW group differ from the OOW in terms of unobservable characteristics that are correlated with EQ-5D, comparing the two groups in a cross-sectional setting will give a biased estimate of the utility gain from transitioning into work. We therefore use the Understanding Society data, which is longitudinal and contains data on both health and work, to estimate the change in utility when individuals move between employment states.

### **3.3.3 Estimating the health utility gain from transitioning into employment**

The Understanding Society (Section 2.5) longitudinal data are used in a regression analysis to estimate the average EQ-5D utility gain from those who move from non-employment into employment in the general population. The equation to be estimated is given in Equation 3, where $U_{it}$ is the health utility of individual $i$ at time $t$ measured by EQ-5D. $U_{it}$ is a function of employment status $EMPL_{it}$ (a binary variable equal to 1 if employed or self-employed, 0 otherwise), and a set of control variables $X_{it}$ which consists of age, sex, index of multiple deprivation (IMD) quintile, marital status, an indicator for if the individual considers themselves to have a disability, and time indicators.

$$U_{it}=\alpha+\beta EMPL_{it}+\delta X_{it}+\mu_{i}+\epsilon_{it} \left( 3 \right)$$

The model contains an error term with two components, ($\mu_{i}+\epsilon_{it}$). The first, $\mu_{i}$, is an individual level time-invariant error and the second is both time and individual-varying, and assumed to be normally distributed with a mean of zero. The fixed-effects estimator applies a time-demeaning transformation to the equation, where for each individual i at time t the mean of the variable over time at the individual level is subtracted:

$$U_{it}-\bar{U}_{i}=\alpha+\beta\left( EMPL_{it}-\bar{EMPL}_{i} \right)+\delta\left( X_{it}-\bar{X}_{i} \right)+\left( \epsilon_{it}-\bar{\epsilon}_{i} \right) \left( 4 \right)$$

$$\ddot{U}_{it}=\alpha+\beta\ddot{EMPL}_{it}+\delta\ddot{X}_{it}+\ddot{\epsilon}_{it} \left( 5 \right)$$

Equation 5 is the time de-meaned equation which eliminates the $\mu_{i}$ term from Equation 3. Under the assumption that all confounding factors correlated with both health and employment are captured in either the observables ($X_{it}$) or the unobserved individual-specific time-invariant error ($\mu_{i}$), Equation 5 can be estimated consistently by Ordinary Least Squares (OLS) to produce an unbiased estimate of the impact of employment on health, $\beta$.This method allows us to observe the mean change in utility observed when a given individual transitions from non-employment into employment.

The Understanding Society data waves 1-13 were used for the analysis. EQ-5D health utilities are not directly available in the Understanding Society data, and so we obtained EQ-5D estimates by mapping from responses to the 12-item Short Form survey, SF-12, using a previously published mapping algorithm^10^. Note that this algorithm is a mapping to the EQ-5D-3L, rather than EQ-5D-5L which is our main measure of health utility. The data were prepared and cleaned, including the mapping from SF-12 to EQ-5D, using the ukhlsclean R package^11^.

We use the estimate of the utility gain from moving into employment from the USoc regressions to estimate the counter-factual health utility for the same OOW population subgroups in the HSE data if they were to gain employment. The resulting utilities are presented in the table below.

| Employment Status | MSK | MH | MSK + MH |
| --- | --- | --- | --- |
| Out-of-work (OOW) | 0.406 | 0.448 | 0.269 |
| In-work (IW) | 0.429 | 0.472 | 0.293 |

Table S3 Estimated EQ-5D utilities by health condition and employment status

These utilities are applied to the number of individuals in the respective health state and employment state at each time point in the model, converted to QALYs by multiplying by 0.25 to adjust the annual figure to quarterly and then multiplied by the number of people in the employment state. To measure the health outcome in financial terms, each QALY is given a value of £70,000 in 2020/21 prices, in line with the Green Book recommendations on programme appraisals which include QALYs^7^.

## **3.4 Modelling Tax and Benefits**

### **3.4.1 UKMOD**

To model the fiscal impact of changes in employment and earnings we use UKMOD^12^, an open-source tax-benefit microsimulation model for the UK maintained by the Centre for Microsimulation and Policy Analysis (CeMPA). The model is based on input data from the Family Resources Survey (FRS). UKMOD version B1.09 is used to model the income, tax, and benefit outcomes. This version was released on 24th November 2023, updated to incorporate the changes announced in the Autumn Statement.

### **3.4.2 Modelling approach**

To model the income, tax, and benefit outcomes for each of the sub-populations of interest we construct an input dataset for UKMOD from the FRS using the [frsclean](https://github.com/djmorris1989/frsclean) R package^13^ version 1.0.2. We then construct three filtered versions of the dataset, one each for the MSK, MH, and MSK+MH sub-populations. Each is then further divided into an in-work (IW) (employees, employers, self-employed) and an out-of-work (OOW) dataset, to model outcomes for both employment states in the cost-benefit analysis for each of the three health conditions.

The six UKMOD model runs are:

- B1.09_MSK_OOW
- B1.09_MH_OOW
- B1.09_MSK_MH_OOW
- B1.09_MSK_IW
- B1.09_MH_IW
- B1.09_MSK_MH_IW

The IW group are modelled as a counterfactual group to estimate the income, tax, and benefit profile of an OOW individual who subsequently gains employment. A simple cross-sectional comparison of those in-work with those out-of-work at a particular point in time is unlikely, however, to provide an accurate estimate of the impact of an OOW individual transitioning into work, as the two populations in the data will differ by both observable and unobservable characteristics.

We therefore filter the IW population further using additional variables in the FRS. We select the subset of the IW group with a particular health condition who have i) been in their current job for less than 365 days (i.e., 1 year), and ii) prior to their current job were not employed. This produces a more relevant counterfactual group to those who are OOW - individuals who are in work but are within a year of having been previously out of work. The additional 3 UKMOD model runs which apply this additional filtering are labelled as:

- B1.09_MSK_IW_1yr
- B1.09_MH_IW_1yr
- B1.09_MSK_MH_1yr

The data used as the input to UKMOD are the pooled FRS datasets for the fiscal years 2018/19 to 2021/22. Data are pooled to maximise the number of potential observations for the IW (<1yr) subgroups, which when combined with filtering by health condition produces small numbers of observations. All monetary variables are adjusted for inflation to 2021 prices using the consumer price index (CPIH). Monetary values are then automatically uprated by UKMOD to 2023 prices when applying the 2023 tax and benefit system.

Figure S4: UKMOD summary outputs for average monthly earnings, benefits, and taxes by health condition and work status

The output of each model run is a detailed breakdown of total market incomes (split between employment and non-employment related income), total benefit payments (separately for each benefit in the model), and total income tax receipts for the population modelled. Each variable is divided by the total subgroup population size to obtain a subgroup average for each variable.

### **3.4.3 Modelled Parameters for Income, Tax, and Benefits**

Figure S4 presents summary output from the UKMOD model runs for average monthly earnings (from employment and self-employment), benefit payments, and direct taxes (defined as income tax plus council tax, not including national insurance contributions).

The horizontal axis indicates the joint health condition / work status and presents results for both the full IW sample and the IW sample restricted to the IW sample who have been in work for less than 1 year and were non-employed prior to their current job. The outcomes of this latter group are used for the tax/benefit/income profiles of those moving from out of work to in-work in the model. For each subpopulation, the sample size from which the estimates were derived is reported. The sample restriction of the IW group leads to a substantial reduction in the estimate of average gross monthly earnings for the IW group for each health condition. The estimate is 69.8% of the full IW sample figure for the MSK group (£1,485 vs £2,126), 67.9% for the MH group (£1,403 vs £2,065), and 59.5% (£1,035 vs £1,738) for the MSK+MH group. The same pattern is observed for income tax receipts, which are substantially lower when using the IW < 1yr group compared to the full IW group due to the smaller estimated average earnings figures.

Correspondingly, the estimated average benefit receipt is larger when considering the IW < 1yr group compared to the full IW sample. In the case of the MH group, average benefit receipt is almost double (£131 vs £66) and is 66% for the MSK+MH group (£166 vs £100). This is also true of the MSK group, though the relative difference is much smaller at 8.7% (£75 vs £69). Higher average benefit receipt is related to lower earnings through increased eligibility for in-work benefits i.e., tax credits. Note that the sample sizes are small for the IW < 1yr groups, particularly for MSK and MSK+MH where the calculations are based on sample sizes of less than 100 across the four pooled FRS datasets.

## **3.5 Other Financial Costs and Benefits**

Table S4 uses employment figures from the DWP’s Social Cost Benefit Analysis Framework^5^, which assumes people move from unemployment to full-time jobs. However, our model displays average weeks worked by quarter. To account for this, the DWP values are adjusted from annual values to weekly. These values are then weighted by average number of weeks worked in a quarter. All values are adjusted for inflation to 2023 prices using the consumer price index (CPIH).

|  | Annual values (Fujiwara 2010) | Annual values (IPS-MOD 2023 prices) |
| --- | --- | --- |
| Reduction in healthcare costs for an individual moving from non-employment to full-time employment | £508 per non-sick benefits individual per year  £1016 per sick benefits individual per year | £742.82 per non-sick benefits individual per year  £1485.65 per sick benefits individual per year |
| Increase in childcare costs for an individual moving from non-employment to full-time employment | £744 per lone parent per year  £694 per couple with children per year | £1087.92 per lone parent per year  £1014.80 per couple with children per year |
| Increase in travel costs for an individual moving from non-employment to full-time employment | £433 per year | £633.16 per year |

Table S4 Summary of other financial costs and benefits included in IPS-MOD

### **3.5.1 Transport costs**

£12.18 is multiplied by the average number of weeks worked for each health condition. This value is an estimate of the additional transport costs an individual faces when moving from non-employment to employment.

### **3.5.2 Childcare costs**

DWP provides two childcare costs. One for couples with children, and a higher cost for lone parents. Parental status split (not a parent, lone parent, couple with children) was found using LFS data for each health condition. We defined for each health condition the proportion of individuals who were lone parents at baseline and assumed this proportion does not change over the five-year duration of the model. This proportionate split was used to weight the cost provided by DWP to find an average increase in childcare costs for an individual moving from non-employment to full-time employment for each health condition.

### **3.5.3 NHS Spending**

DWP provides two costs for reductions to healthcare costs. A higher cost for those with disabilities who would participate in Employment and Support Allowance (ESA) programmes, and a lower cost for those who wouldn’t. HLET data provides the proportion of recruits to the treatment group that are ESA claimants. This split was used to weight the costs provided by DWP to find an average reduction to healthcare costs for an individual moving from non-employment to full-time employment for each health condition that would be representative of a population likely to uptake IPS.

Moving those non-employed into work will increase their health utility and therefore reduce demand for healthcare services. Individuals are eligible for sick benefits if they have a disability or health condition that affects how much they can work, therefore, healthcare service utilisation is likely to be greater for these individuals. Meaning, the reduction in healthcare costs from gaining employment will be greater for those claiming sick benefits than for those who are not.

The difference is healthcare utilisation is based on the likelihood that those who receive sick benefits are likely to have a greater prevalence of health conditions than those who do not receive sick benefits. However, given that our subgroups of interest are those with health conditions it is possible that the model underestimates the reduction in healthcare costs associated with moving into employment.

| Health condition | Increase in childcare costs | Increase in travel costs | Reduction in health costs |
| --- | --- | --- | --- |
| MSK | £4.17 | £12.18 | -£15.90 |
| MH | £5.19 | £12.18 | -£15.90 |
| MSK + MH | £3.95 | £12.18 | -£15.90 |

Table S5 Summary of changing costs per week of moving from non-employment to full-time employment by health condition

## **3.6 Modelling labour market transitions**

We model the labour market transitions of the target populations over five years using the LFS (Section 2.6) longitudinal data. We use 27 longitudinal datasets covering starting quarters in wave 1 of Q2 2012 to Q4 2018. These datasets cover the period of time for which health condition information is available in the longitudinal LFS dataset releases.

The longitudinal data in the LFS track individuals for five quarters, interviewing respondents at 13-week intervals. Wave 1 is used to define the model baseline (time $t=0$ in the model), to identify the population of interest (working age, non-employed, and report MSK and/or MH conditions) and their characteristics. Note that in the LFS individuals are asked to report health conditions specifically which impact their ability to work. Consequently, the data which we use to calculate the transition probabilities will exclude individuals who have MSK and/or MH conditions if those individuals do not consider that their health conditions impact their ability to work. MH subgroup is filtered to individuals who report a MH condition. MSK subgroup is filtered to individuals who report a MSK condition. MSK+MH subgroup is filtered to individuals who report both a MH and a MSK condition.

The data from all waves of the pooled longitudinal LFS data are used to determine quarterly transitions between employment and non-employment for each sub-population of interest for the first year of the model ($t=1-4$). For these first four quarters after baseline, transition probabilities are allowed to vary based on duration of employment e.g. individuals who have been in employment for three previous quarters can have a different probability of becoming non-employed than those who have been employed in only one previous quarter. For years 2 to 5 ($t=5-20$) we extrapolate further transitions in employment status using the probabilities derived from the data.

Labour market transitions are simulated separately for each of the three health conditions represented in the model. As the LFS contains information on the type of social security benefits received by those out of work, for each condition we separately model a “SRB” and “non-SRB” set of labour market transitions, to separately model the transitions of those in receipt of Sickness Related Benefits (SRB). SRB is defined at baseline (i.e. in the first quarter surveyed). The benefits included in this definition are all benefits for which not being employed and having a work-limiting health condition(s) are part of the eligibility criteria; Universal Credit (for those who are sick/disabled), Income Support (for illness/disability), Employment and Support Allowance (ESA), Incapacity Benefit, and Severe Disablement Allowance.

### **3.6.1 Year One**

For year one we are able to model detailed labour market transition probabilities which reflect the previous employment history of the individual. For example, for a group of individuals who are observed in employment in wave 4 of the data, some may have been out of work for the duration of waves 1, 2, and 3, whereas others may have been in work in waves 2 and 3. We can therefore calculate transition probabilities which vary according to the duration of employment.

Figure S5: Example transition probability tree for year one of the labour market simulation

This produces a “tree” of potential labour market transitions for year one of the labour market simulation, which Figure S5 illustrates for the case of musculoskeletal conditions. There are 16 possible end states by the end of year one, which defines every combination of non-employment and employment that can be observed for an individual at the four time points observed after the baseline from “UUUU” (never observed in employment) to “EEEE” (employed consistently from wave 2 onwards).

#### **3.6.1.1 Employment durations**

##### **3.6.1.1.1 For those employed**

Note that so far this method is only informative about employment status at four distinct points in time evenly spaced throughout the year. We cannot assume that, for example, an individual observed as employed in the week they were interviewed in wave 3 has been consistently employed for the full preceding quarter since interviewed in wave 2. We therefore calculate at each node in the tree the average amount of weeks worked in the previous quarter.

The LFS collects data on the specific week each participant completes the survey (1-13). This variable was translated into months across the five waves of data (1-15). This was combined with the variable on the employment status indicator variable, to create a variable of the month when a participant said they were employed or non-employed.

The LFS also collects data on the month participants began their current job. This variable was adjusted so that month 1, was the first month of the dataset, and 15 was the last month. As wave 1 is filtered so everyone is unemployed. Each individual can only work for a maximum of 12 months. For individuals who were employed between survey dates, months worked in each wave were calculated as the difference between the month a participant answered if they were employed or not and the month they started their current job.

Some individuals had data that they worked within a certain wave, but no data on when they answered the survey and/or the month they began their current job. For these individuals, we imputed values of average weeks worked for those employed in the given wave. This was calculated for each health and sickness benefits condition at each wave in the tree.

##### **3.6.1.1.2 For the non-employed**

An individual reporting being non-employed at the survey date may still have had spells of employment in the preceding quarter.

At each survey wave the LFS contains a variable indicating the month when a participant left their most recent job, in 3-month increments. To capture employment within the wave, this variable was converted into a dummy variable. A value of 1 indicates the participant reported working in the past 3 months (within the wave), while 0 indicates otherwise.

As this variable is in 3-month increments, we cannot use it to estimate an average weeks value (as each wave is 3 months). To estimate weeks worked for in instances like these, we looked at data on weeks worked from participants who were employed in a given wave, but sandwiched by waves of non-employment. I.e.:

- UEU
- UUEU
- UEUEU
- UUUEU

This data was combined to find average weeks worked value for participants who were non-employed on the survey date but left their last job within the wave. This was calculated for each health and sickness benefits condition at each wave in the tree.

The average weeks worked is a value between 0 and 13. For all nodes, this number will lie between the two because even between two nodes with the same employment status (E->E or U->U) there may be some transitory employment / unemployment of short spells which are missed by the quarterly reporting of current employment status e.g. someone may have been employed in wave 2, lose their job, and then find another job within a month.

### **3.6.2 Years Two to Five**

To project outcomes over a five-year period, we developed a Markov transition probability model. This model allows us to predict the likelihood of people moving between employment and non-employment, including those remaining in their current state (employed or non-employed) for the next quarter (13 weeks). The Markov model was constructed from LFS data for each health condition. While the Markov model is similar to the tree structure used for the year-one analysis, it is simplified in that probabilities no longer depend on previous employment history.

Figure S6: Markov model structure

To calculate the likelihood of someone transitioning from non-employment to employment, we analysed data from four waves of the LFS. We focused on individuals who were non-employed in the previous wave and then calculated the proportion of those who became employed in the subsequent wave. This is the transition probability of moving from non-employment to employment. Subtracting this value from 1 gives us the probability of someone staying non-employed.

The average number of weeks worked in a quarter (13 weeks) was calculated. Averages were calculated across the 4 waves of data available. Separate averages were determined for individuals based on employment status, health condition, and the status of claiming sickness benefits.

|  | Average weeks worked | | | |
| --- | --- | --- | --- | --- |
|  | Non-Disabled/ not claiming sick benefits | | Disabled/ claiming sick benefits | |
|  | Non-Employed | Employed | Non-Employed | Employed |
| Low to moderate musculoskeletal health conditions | 0.44 | 11.86 | 0.01 | 11.88 |
| Low to moderate mental health conditions | 0.56 | 11.86 | 0.01 | 12.12 |
| Low to moderate mental health conditions AND low to moderate musculoskeletal conditions | 0.31 | 11.87 | 0.00 | 10.95 |

Table S6 Average Weeks Worked per Quarter by Employment Status, Health Condition, and Benefit Status

These values were used to find a population average of weeks worked in a quarter. This was found by seeing the proportion of people in ‘employed’ and ‘non-employed’ at each quarter for each health condition group for each status of claiming sickness benefits.

# **4. Modelling the IPS intervention and control scenarios**

This section describes how the baseline model outlined previously is configured to model the effect of IPS. It does so through constructing a control arm in which no-one receives the intervention, and an intervention arm in which all eligible individuals receive the intervention. IPS-MOD allows for the IPS intervention to produce work and health related outcomes through two key mechanisms:

1. Transition probabilities. IPS makes transitions to (or remaining in) non-employment less likely. The intervention therefore increases employment and so income, income tax payments, and employment-attributable health. It also reduces social security payments (Section 4.1).
2. Health utility. Receipt of the IPS intervention has a positive impact on health-related quality of life. Regardless of employment status, receiving the intervention will increase health utility. The analysis described so far is based on the transition probabilities that were estimated from the LFS, from a full working-age population of individuals who begin non-employed and have one or both of musculoskeletal and mental health conditions.

In the Health Led Employment trials, the labour market outcomes in both the control and treatment groups were better than those obtained by modelling the LFS. Therefore, to compare the IPS intervention outcomes to the LFS data would over-estimate the true treatment effect and inflate the estimate of the return on investment. We therefore construct both a control arm and an intervention arm in the model based on calibrating parameters from the business-as-usual (BAU) arm to reproduce the results of the trial. The three arms modelled by IPS-MOD are, in summary:

1. Business-as-usual (BAU): The outcome of modelling the transition probabilities obtained from Labour Force Survey data analysis.
2. Control/No Intervention arm (Control): Replicating the BAU arm with calibration factors applied to transition probabilities such that the model reproduces the HLE trial control arm 12-month outcomes.
3. IPS/Intervention arm (Intervention): Replicating the BAU arm with calibration factors applied to transition probabilities such that the model reproduces the HLE trial intervention arm 12-month outcomes.

The latter two of the these arms are used by the model to calculate the treatment effects.

## **4.1 Adjusting transition probabilities for treatment effects**

### **4.1.1 Year One**

The intervention and control arms are constructed by calibrating the probabilities of non-employment in the BAU arm to match outcomes from the HLE Trials Evaluation control and treatment arms. There are two key outcomes which are used to calibrate the transition probabilities; the percentage of individuals in work at 12 months after receiving the intervention, and the percentage of individuals who gained employment at any point in the 12 months following the intervention.

Below we report these outcomes from the baseline LFS data, and the two arms of the HLE Trials. Note that the outcomes from the HLE Trials was calculated as a weighted average of the outcomes of the Sheffield City Region out of work participants, and the West Midlands Combined Authority participants.

| Outcome measure | BAU (Labour Force Survey) | HLE Trials Control | HLE Trials Intervention |
| --- | --- | --- | --- |
| % in work at 12 months post-intervention | 13.08% | 19.86% | 23.55% |
| % in work at any point during 12 months post-intervention | 15.10% | 31.52% | 36.97% |

Table S7 Labour market outcomes used for model calibration

**Step 1: Calibrate the employment rate “at 12 months”**

The model is first calibrated to the “in work at 12 months post-intervention” outcome. This is because this is the outcome which can be most accurately calculated in the LFS data. The calibration is performed by applying multipliers to the non-employment transition probabilities, which alter the distribution of transitions through the nodes of the tree represented in Figure S5.

Calibration factors are created that reduce the probability of being non-employed relative to the BAU arm. There are four calibration factors which impact different probabilities categorised by the duration of employment:

| Factor Label | Description | Calibration Factor (Control) | Calibration Factor (Intervention) |
| --- | --- | --- | --- |
| U-U | Applied to all probabilities of remaining non-employed. | 0.9833 | 0.9700 |
| E1-U | Applied to all probabilities of becoming non-employed for those who have one quarter of employment. | 0.1931 | 0.0391 |
| E2-U | Applied to all probabilities of becoming non-employed for those who have two consecutive quarters of employment. | 0.7086 | 0.4297 |
| E3-U | Applied to all probabilities of becoming non-employed for those who have two consecutive quarters of employment. | 0.9925 | 0.5314 |

Table S8 Transition probability calibration factors

Calibration factors range from 1 (no adjustment to probabilities from their BAU/LFS baseline values), to 0 (100% chance of entering employment). The calibration factors are therefore interpreted as relative treatment effects vs the BAU arm. Note that as the HLE trial only reports outcome measures for the whole OOW group, with no dis-aggregation by health condition or the status of claiming sickness benefits. The calibration is performed for the population which is distributed across health conditions and the status of claiming sickness benefits in the same proportions as in the trial and the same calibration factors are applied to each individual health condition / sickness benefit receipt status specific tree.

| MH | 36.6% |
| --- | --- |
| MSK | 11.5% |
| MSK+MH | 51.8% |
| Claiming Sickness Related benefits | 11% |

Table S9 Trial Demographic

Calibration factor values were determined by simulating the key outcomes resulting from combinations of randomly drawn calibration factors in R. Results of the simulation were then filtered to a set of conditions in order to arrive at the factors applied in the model. Calibration factors were filtered to produce a result within 0.5% of the ‘% in work at the 12-month point’ for both control and intervention groups. The four calibration factors are selected to ensure they individually produced a smaller effect on the control group compared to the intervention group, to ensure the HLE trial finding that IPS is effective is replicated. Calibrations were further filtered such that the effectiveness of IPS diminishes monotonically over time.

**Step 2: Calibrate the employment rate “at any point within 12 months”**

This approach yields calibration factors which, when reproducing the % in-work **at** 12 months outcome from the trial, tends to under-estimate the % in-work **at any point** in 12 months. The reason for this under-estimation is because of the discrete time points at which individuals are surveyed in the LFS. The employment status is recorded only for the survey week, and therefore those who report being non-employed in each of the 5 waves of the LFS could potentially have had employment spells of short duration during the 12 weeks between survey dates. The “never employed” branch (the top branch in Figure S5) of the trees therefore will underestimate employment among these individuals.

We therefore apply an adjustment to the number of individuals on the “never employed” branch such that there is a non-zero number who have had some employment over the 12-month period but which was not observed at the point of the survey. Firstly, we estimate this number for the BAU arm by using a variable in the LFS which asks non-employed individuals when they left their last job. The number of individuals in the “never employed” branch who answer less than 12 months to this question in the wave 5 survey is the BAU estimate of the number of those individuals never **observed** to be in employment during their participation in the survey, but did have some employment of short duration between survey dates. This is estimated to be 0.56%.

In the intervention and control arms, this 0.56% figure is calibrated such that the “% in work at any point in 12 months” outcome matches the outcome for the respective arm of the HLE trial. This calibration step is taken conditional on having applied the calibration factors for the “% in work at 12 months” outcome described above. This calibration raises the proportion of those in the “never employed” branch who were employed at some point in the 12 months but not being observed to be so. The resulting employment rates for this branch of the trees which are consistent with the HLE trial outcomes are 13.51% and 15.90% for the control and intervention arms respectively.

### **4.1.2 Years Two to Five**

The HLE trial does not have outcomes for the full five-year time horizon which is applied in IPS-MOD. Modelling beyond year one consequently requires making assumptions about the persistence of the treatment effect of IPS on individuals’ probabilities of non-employment. The model includes four possible scenarios for this which are illustrated in Figure S7:

1. IPS full effect Year 1 then diminishes to Control in Year 5
2. IPS leads to a permanent/structural improvement
3. IPS full effect until Year 2 then diminishes to Control in Year 5
4. IPS full effect until Year 3 then diminishes to Control in Year 5

The model default is to assume scenario 2 - the treatment effect of IPS on the non-employment probabilities diminishes linearly over years 2-5 until year 5 in which it remains at 25% of its year 1 value. The model allows the user to choose from among these four scenarios in order to conduct deterministic sensitivity analysis of the results to the assumption about treatment effect persistence. While there are other potential scenarios e.g. treatment effect remains at 100% of year 1 value indefinitely, or diminishes immediately to 0% in year 2, these were not considered credible and so are not included in the model as options.

Figure S7: Different scenarios for the effect of IPS in years 2-5 post-receipt relative to the year 1 effects

## **4.2 Adjusting health utilities for treatment effects**

The gains in health from an employment programme have both a direct and indirect component. The indirect health benefits come from higher employment rates, as a greater proportion of the population in work as a result of IPS will generate more QALYs than without via the health effects of employment estimated in Section 3.3. The direct effect is a direct impact of IPS on health itself, independent of changes in employment status.

The HLE trials^1^ published outcomes on the overall health utility gains from IPS incorporating both of these effects. The average gains to health utility for the combined out of work group was a 0.02 QALY gain per individual. With just the indirect effects included in the model and the population set to the same distribution across health conditions as in the trial, the gain is 0.0025 - approximately one tenth of the total effect estimated by the HLE.

The total health utility gains from transitioning into employment estimated from the USoc data analysis are calibrated to replicate this outcome. To do this we apply a constant multiplier to each OOW and IW health utility for each health condition which replicates the same total QALY gain of 0.02 that was reported in the trial. This multiplier is 1.0155. The IPS intervention arm of the model applies the control arm utilities multiplied by 1.0155 as the health utilities for each health/employment state.

| Employment Status | MSK | MH | MSK+MH |
| --- | --- | --- | --- |
| ***Control Arm*** |  |  |  |
| Out-of-work (OOW) | 0.406 | 0.448 | 0.269 |
| In-work (IW) | 0.429 | 0.472 | 0.293 |
|  |  |  |  |
| ***Intervention Arm*** |  |  |  |
| Out-of-work (OOW) | 0.411 | 0.454 | 0.272 |
| In-work (IW) | 0.435 | 0.478 | 0.297 |

Table S10 IPS-MOD health utilities by health condition, employment, and arm

## **4.3 Allowing for differential performance**

A key function of the model is to produce CBA for appraisal of local implementations of an IPS programme. These implementations will have their own costs and 12-month performance metrics which differ from the HLE trial outcomes which were used to calibrate the model. IPS-MOD allows the user to input their own observed employment rate within 12 months, which will adjust the calibration factors applied to the transition probabilities to re-calibrate the intervention arm of the model to the supplied metric.

This is implemented by applying a multiplier, $m$, to each of the four calibration factors, $c$. The model includes a lookup table which, for each integer percentage from 0% to 100%, returns the multiplier which, when applied to the calibration factors for the intervention arm, replicates the selected % of individuals who obtained employment within the first 12 months of receiving the intervention.

The underlying multiplier adjusts the calibration factor by the difference between 1 and the calibration factor if the input is a worse outcome than that of the HLE intervention arm. The adjustment is made to the difference between the calibration factor and 0 if the input is a better outcome than the HLE intervention arm.

Figure S8: Calibration factors for each employment rate - MSK & MH

- $c'=c+\left( 1-c \right)m$ if $m>0$
- $c'=c+\left( c-0 \right)m$ if $m<0$

The multiplier $m$ is scaled such that it takes on a value of -1 to 1, where:

- 1 adjusts the calibration factor to 1, i.e. the BAU/LFS outcome.
- 0 applies no adjustment, produces the HLE treatment arm outcome.
- -1 adjusts the calibration factor to 0, i.e. 100% probability of employment.

Note that the multiplier constrains the worst possible outcome to equal that of the BAU arm. While it is technically possible for employment rates within 12 months to be worse than the BAU outcome, these values are assumed to be implausible. Any user input provided which is worse than the BAU outcome is automatically adjusted upwards by the model to equal the BAU employment at any point in 12 months outcome. Multipliers are generated separately for each of the three health condition options and for each proportion of individuals in the population in receipt of out of work disability benefits. Figure S8 illustrates the adjustment of the calibration factors relative to the specified employment rate at 12 months for those with MSK and MH conditions.

## **4.4 Cost-Benefit Analysis**

The cost benefit analysis is conducted through a societal perspective, in line with the Green Book^7^ recommendations. Total net benefits (total benefits minus total costs), as well as government gains and individual gains are also calculated. Costs and benefits were calculated by comparing the averages of the intervention and control arms. Costs and benefits are then presented as individual-level values and upscaled to the whole population.

The BCR is calculated for the societal perspective by dividing the societal perspective total benefits by total costs. The interpretation of the BCR is the amount received in benefits per £1 spent on IPS, with a BCR of one representing break-even and a BCR above one representing a positive return on investment e.g. A BCR of 1.10 indicates that for every £1 spent on IPS society receives £1.10 in benefits.

| **Societal perspective benefit cost ratio** | |
| --- | --- |
| **Benefits** | **Costs** |
| Savings in National Health Service (NHS) spending | Cost of IPS |
| Increase to wages |  |
| Value of gains to health utility |  |
| Change in childcare |  |
| Change in transport |  |
| **Government gains** | |
| **Benefits** | **Costs** |
| Savings in National Health Service (NHS) spending |  |
| Savings in social security payments |  |
| Increases in direct tax (income tax + council tax) |  |
| Increases in employee/self-employed national insurance |  |
| Increases in employer national insurance |  |
| **Individual gains** | |
| **Benefits** | **Costs** |
| Value of gains to health utility | Increases in direct tax (income tax + council tax) |
| Increase to wages | Increases in employee/self-employed national insurance |
| Savings in social security payments | Change in childcare costs |
|  | Change in transport costs |

Table S11 Benefits and Costs, societal perspective benefit cost ratio, government gains and individual gains

## **4.5 IPS-MOD Inputs and Outputs**

The final IPS-MOD tool is an interactive modelling tool designed to estimate the economic and health impacts of delivering IPS services within a specified local context. Users can enter a range of key inputs, including: the local authority of interest; the health condition being modelled; the size of the working-age population (16–64 years); the number of people with the specified health condition; the number who are non-employed with that condition; the number who would uptake IPS; the percentage of the population claiming sickness-related benefits; the total service cost; the monetary value of a quality-adjusted life year (QALY); discount rates; the Year 2–5 scenario; and the assumed service effectiveness. Based on these inputs, the tool produces outputs including: the number and percentage of participants gaining employment; the average number of weeks in which paid work took place (per person); the associated change in health utility; the societal perspective benefit–cost ratio; the exchequer gains; the average financial gain per person; and a sensitivity analysis to test the robustness of results. All inputs and outputs are presented in Table S12.

| **IPS-MOD** | |
| --- | --- |
| **Inputs** | **Outputs** |
| - Local authority - Health condition - Working age population (16 - 64) - Population with specified health condition - Non-employed population with specified health condition - Non-employed population with specified health condition who would uptake IPS - % of population claiming sickness related benefits - Service cost - Value of a QALY - Discount rates - Year 2-5 scenario - Service effectiveness. | - Employed (n) - Employed (%) - Average weeks in which paid work took place (per person average) - Health utility change - Societal perspective benefit cost ratio - Exchequer gains - Financial gains (per person average). - Sensitivity analysis |

Table S12 IPS-MOD Inputs and Outputs

# **Part B: Supplementary Results**

# **5. Introduction**

This section presents additional figures and tables to further illustrate the modelled IPS-MOD scenarios and to support the interpretation of the main results of the IPS-MOD analysis presented in the article.

## **5.1 Base case Results for Population of People Living with Mental Health Conditions**

Table S13 first presents employment effects for the MH group. A total of 4,444 participants were included. At baseline (Year 0), neither the IPS nor the non-IPS group were in employment. By the end of Year 1, the employment rate for MH participants receiving IPS services rose to 27.27%, compared with 22.08% for those without IPS services. This represents a 5.19% absolute increase, corresponding to a 23.52% relative gain. By Year 5, the IPS group reached an employment rate of 61.34%, compared to 59.17% for the non-IPS group, reflecting a 2.16% absolute gain and a 3.66% relative gain. In terms of work participation, by the end of Year 1 MH participants in the IPS group had worked an average of 12.49 weeks, 1.46 weeks more than the 11.03 weeks averaged by the non-IPS group. By Year 5, those receiving IPS services had worked an average of 150.27 weeks, an increase of 4.63 weeks over the 145.64 weeks for the non-IPS group.

Health utility, measured in QALYs, improved for the IPS group, with average gains of 0.03 QALYs by the end of Year 1 and 0.150 QALYs by the end of Year 5. The corresponding monetary value of these QALY gains was £2,435.55 per person by Year 1 and £11,937.53 per person by Year 5. Aggregated across the MH population, these gains equated to £10.823 million by Year 1 and £53.048 million by Year 5.

Table S13 also shows the financial impact for government and individuals in the MH group. For the public sector, savings in NHS spending were £99,059.35 by the end of Year 1 and £305,835.79 by Year 5. Savings in social security payments reached £744,883.19 by Year 1 and £2,299,752.06 by Year 5. Additional government revenue came from increases in direct tax, employee/self-employed national insurance, and employer national insurance contributions. Total benefits to government amounted to £1.300 million by Year 1 and £4.014 million by Year 5. For individuals in the IPS group, cumulative gains averaged £2,585.45 by Year 1 and £12,400.31 by Year 5.

From a societal perspective, the benefit-cost ratio indicated that every £1 spent on IPS returned £1.22 by the end of Year 1 and £5.67 by the end of Year 5. Table S15 presents a breakdown of the components of the societal perspective benefit cost ratio as well as the effect IPS has on taxes and social security payments on an individual level. In summary, the IPS service improved employment outcomes, increased weeks of paid work, enhanced health utility, and yielded significant economic benefits from both individual and societal perspectives over the five-year period for the MH group.

|  | **Year 0** | **By the end of year 1** | **By the end of year 5** |  |
| --- | --- | --- | --- | --- |
| **Probability of Being Employed (%)** | | | |  |
| Without IPS service | 0.00% | 22.08% | 59.17% |  |
| With IPS service | 0.00% | 27.27% | 61.34% |  |
| Gains from IPS (absolute change) | 0.00% | 5.19% | 2.16% |  |
| Gains from IPS (% change) | 0.00% | 23.52% | 3.66% |  |
| **Average weeks in which paid work took place per person** | | | |  |
| Without IPS service | 0 | 11.03 | 145.64 |  |
| With IPS service | 0 | 12.49 | 150.27 |  |
| Gains from IPS | 0 | 1.46 | 4.63 |  |
| **Health utility change** | | | |  |
| QALY gains to those receiving IPS service (per person average) | | 0.03 | 0.150 |  |
|  |  |  |  |  |
| Value of QALY gains to those receiving IPS service (£) (per person average) | | £2,435.55 | £11,937.53 |  |
|  |  |  |  |  |
| Value of QALY gains to those receiving IPS service (Million £) (population total) | | £10.823M | £53.048M |  |
|  |  |  |  |  |
| **Costs and benefits to government (population total)** | | | |  |
| Savings in National Health Service (NHS) spending | | £99,059.35 | £305,835.79 |  |
|  |  |  |  |  |
| Savings in social security payments | | £744,883.19 | £2,299,752.06 |  |
|  |  |  |  |  |
| Increases in direct tax (income tax + council tax) | | £202,732.73 | £625,916.95 |  |
|  |  |  |  |  |
| Increases in employee/self-employed national insurance | | £103,147.36 | £318,457.10 |  |
|  |  |  |  |  |
| Increases in employer national insurance | | £150,293.26 | £464,015.34 |  |
|  |  |  |  |  |
| Total Benefits (Million £) | | £1.300M | £4.014M |  |
|  |  |  |  |  |
| **Individual gains (per person average)** | | | |  |
| Cumulative gains (£) to those on IPS service | | £2,585.45 | £12,400.31 |  |
| **Societal perspective benefit cost ratio** | | | |  |
| Every £1 spent on IPS service returns | | £1.22 | £5.67 |  |
|  |  |  |  |  |

Table S13: IPS-MOD Results for a typical local authority for Mental Health Conditions (4444 Participants). Effects on employment, health, exchequer, and individual income.

## **5.2 Base case Results for Population of People Living with Mental Health and Musculoskeletal Conditions**

Table S14 first presents employment effects for the MSK + MH group. A total of 1,487 participants were included. At baseline (Year 0), neither the IPS nor the non-IPS group were in employment. By the end of Year 1, the employment rate for MSK + MH participants receiving IPS services rose to 22.86%, compared with 18.49% for those without IPS services. This represents a 4.37% absolute increase, corresponding to a 23.62% relative gain. By Year 5, the IPS group reached an employment rate of 60.90%, compared to 56.55% for the non-IPS group, reflecting a 4.36% absolute gain and a 7.70% relative gain. In terms of work participation, by the end of Year 1 MSK + MH participants in the IPS group had worked an average of 7.24 weeks, 1.38 weeks more than the 5.86 weeks averaged by the non-IPS group. By Year 5, those receiving IPS services had worked an average of 107.81 weeks, an increase of 10.24 weeks over the 97.57 weeks for the non-IPS group.

Health utility, measured in QALYs, improved for the IPS group, with average gains of 0.0186 QALYs by the end of Year 1 and 0.0939 QALYs by the end of Year 5. The corresponding monetary value of these QALY gains was £1,478.90 per person by Year 1 and £7,463.94 per person by Year 5. Aggregated across the MSK + MH population, these gains equated to £2.199 million by Year 1 and £11.097 million by Year 5.

Table S14 also shows the financial impact for government and individuals in the MSK + MH group. For the public sector, savings in NHS spending were £31,353.47 by the end of Year 1 and £217,532.23 by Year 5. Savings in social security payments reached £253,311.32 by Year 1 and £1,757,488.85 by Year 5. Additional government revenue came from increases in direct tax, employee/self-employed national insurance, and employer national insurance contributions. Total benefits to government amounted to £0.365 million by Year 1 and £2.533 million by Year 5. For individuals in the IPS group, cumulative gains averaged £1,561.49 by Year 1 and £8,037.01 by Year 5.

From a societal perspective, the benefit-cost ratio indicated that every £1 spent on IPS returned £0.77 by the end of Year 1 and £4.13 by the end of Year 5. Table S16 presents a breakdown of the components of the societal perspective benefit cost ratio as well as the effect IPS has on taxes and social security payments on an individual level. In summary, the IPS service improved employment outcomes, increased weeks of paid work, enhanced health utility, and yielded significant economic benefits from both individual and societal perspectives over the five-year period for the MSK + MH group.

|  | **Year 0** | **By the end of year 1** | **By the end of year 5** |  |
| --- | --- | --- | --- | --- |
| **Probability of Being Employed (%)** | | | |  |
| Without IPS service | 0.00% | 18.49% | 56.55% |  |
| With IPS service | 0.00% | 22.86% | 60.90% |  |
| Gains from IPS (absolute change) | 0.00% | 4.37% | 4.36% |  |
| Gains from IPS (% change) | 0.00% | 23.62% | 7.70% |  |
| **Average weeks in which paid work took place per person** | | | |  |
| Without IPS service | 0 | 5.86 | 97.57 |  |
| With IPS service | 0 | 7.24 | 107.81 |  |
| Gains from IPS | 0 | 1.38 | 10.24 |  |
| **Health utility change** | | | |  |
| QALY gains to those receiving IPS service (per person average) | | 0.0186 | 0.0939 |  |
|  |  |  |  |  |
| Value of QALY gains to those receiving IPS service (£) (per person average) | | £1,478.90 | £7,463.94 |  |
|  |  |  |  |  |
| Value of QALY gains to those receiving IPS service (Million £) (population total) | | £2.199M | £11.097M |  |
|  |  |  |  |  |
| **Costs and benefits to government (population total)** | | | |  |
| Savings in National Health Service (NHS) spending | | £31,353.47 | £217,532.23 |  |
|  |  |  |  |  |
| Savings in social security payments | | £253,311.32 | £1,757,488.85 |  |
|  |  |  |  |  |
| Increases in direct tax (income tax + council tax) | | £32,297.35 | £224,080.91 |  |
|  |  |  |  |  |
| Increases in employee/self-employed national insurance | | £17,781.90 | £123,371.87 |  |
|  |  |  |  |  |
| Increases in employer national insurance | | £30,337.00 | £210,479.88 |  |
|  |  |  |  |  |
| Total Benefits (Million £) | | £0.365M | £2.533M |  |
|  |  |  |  |  |
| **Individual gains (per person average)** | | | |  |
| Cumulative gains (£) to those on IPS service | | £1,561.49 | £8,037.01 |  |
| **Societal perspective benefit cost ratio** | | | |  |
| Every £1 spent on IPS service returns | | £0.77 | £4.13 |  |
|  |  |  |  |  |

Table S14: IPS-MOD Results for a typical local authority for **Mental Health and Musculoskeletal Conditions** (1487 Participants). Effects on employment, health, exchequer, and individual income.

|  | **Year 1** | **By the end of year 2** | **By the end of year 3** | **By the end of year 4** | **By the end of year 5** |
| --- | --- | --- | --- | --- | --- |
| **Costs (individual level)** | | | | | |
| **Total cost of IPS** | **£2,326.00** | **£2,326.00** | **£2,326.00** | **£2,326.00** | **£2,326.00** |
| **Benefits (individual level)** | | | | | |
| A) Savings in National Health Service (NHS) spending | £22.29 | £51.64 | £68.51 | £73.51 | £68.82 |
| B) Savings in social security payments | £167.62 | £388.30 | £515.20 | £552.75 | £517.51 |
| C) Increase to wages | £410.76 | £951.52 | £1262.50 | £1354.52 | £1268.17 |
| D) Value of gains to health utility | £2,435.55 | £4,887.80 | £7,292.89 | £9,642.67 | £11,937.53 |
| E) Change in childcare | -£7.29 | -£16.89 | -£22.41 | -£24.04 | -£22.51 |
| F) Change in transport | -£17.12 | -£39.65 | -£52.61 | -£56.45 | -£52.85 |
| G) Increases in direct tax (income tax + council tax) | £45.62 | £105.68 | £140.22 | £150.44 | £140.85 |
| H) Increases in employee/self-employed national insurance | £23.21 | £53.77 | £71.34 | £76.54 | £71.66 |
| I) Increases in employer national insurance | £33.82 | £78.35 | £103.95 | £111.53 | £104.42 |
| **J) Societal perspective total benefits**  **(a + c + d + e + f)** | **£2,844.19** | **£5,834.42** | **£8,548.88** | **£10,990.21** | **£13,199.16** |
| **Net benefit** | **£518.19** | **£3,508.42** | **£6,222.88** | **£8,664.21** | **£10,873.16** |
| **Benefit cost ratio (BCR)**  **(J divided by Total costs)** | **£1.22** | **£2.51** | **£3.68** | **£4.72** | **£5.67** |

Table S15 IPS-MOD Results for a typical local authority for **Mental Health Conditions**. Breakdown of societal perspective benefit cost ratio (BCR) by items of benefit and by years.

|  | **Year 1** | **By the end of year 2** | **By the end of year 3** | **By the end of year 4** | **By the end of year 5** |
| --- | --- | --- | --- | --- | --- |
| **Costs (individual level)** | | | | | |
| **Total cost of IPS** | **£2,326.00** | **£2,326.00** | **£2,326.00** | **£2,326.00** | **£2,326.00** |
| **Benefits (individual level)** | | | | | |
| A) Savings in National Health Service (NHS) spending | £21.09 | £56.13 | £92.07 | £122.90 | £146.31 |
| B) Savings in social security payments | £170.37 | £453.52 | £743.84 | £992.97 | £1,182.06 |
| C) Increase to wages | £308.10 | £820.14 | £1,345.17 | £1,795.70 | £2,137.64 |
| D) Value of gains to health utility | £1,478.90 | £2,996.55 | £4,511.43 | £6,003.75 | £7,463.94 |
| E) Change in childcare | -£5.26 | -£14.00 | -£22.96 | -£30.65 | -£36.48 |
| F) Change in transport | -£16.19 | -£43.10 | -£70.70 | -£94.38 | -£112.35 |
| G) Increases in direct tax (income tax + council tax) | £21.72 | £57.82 | £94.84 | £126.60 | £150.71 |
| H) Increases in employee/self-employed national insurance | £11.96 | £31.84 | £52.22 | £69.70 | £82.98 |
| I) Increases in employer national insurance | £20.40 | £54.31 | £89.08 | £118.92 | £141.57 |
| **J) Societal perspective total benefits**  **(a + c + d + e + f)** | **£1,786.64** | **£3,815.73** | **£5,855.01** | **£7,797.33** | **£9,599.06** |
| **Net benefit** | **-£539.36** | **£1,489.73** | **£3,529.01** | **£5,471.33** | **£7,273.06** |
| **Benefit cost ratio (BCR)**  **(J divided by Total costs)** | **£0.77** | **£1.64** | **£2.52** | **£3.35** | **£4.13** |

Table S16 IPS-MOD Results for a typical local authority for **Mental Health and Musculoskeletal Conditions**. Breakdown of societal perspective benefit cost ratio (BCR) by items of benefit and by years.

## **5.3 Sensitivity Analysis**

Tables S16 and S17 show the outcomes of the sensitivity analysis conducted on IPS-MOD for the MH and MSK + MH population, respectively. It presents the BCR under different scenarios of service cost and effectiveness. Negative ROI is indicated by red results; for instance, a BCR of 0.70 means that society receives £0.70 in benefits for every £1 spent on IPS, indicating a negative return on investment. Borderline values are shown in orange, while positive return outcomes are highlighted in green. The analysis demonstrates that economic outcomes are sensitive to both reductions in effectiveness and increases in service cost, with some scenarios resulting in negative ROI in the short term. Results show that for the MH group, IPS delivers a positive return on investment in most modelled scenarios by the end of year 1, and by the end of year 5, all modelled scenarios result in a positive return on investment. Results show that for the MSK + MH group, IPS does not deliver a positive return on investment in most modelled scenarios by the end of year 1, including the central estimate. However, by the end of year 5, almost all modelled scenarios result in a positive return on investment. Further sensitivity analyses are presented in Table S18 and Figures S10 and S11, including the BCR by the end of year 5 under alternative assumptions about the duration of effect, as well as changes in the BCR based on different valuations of a QALY.

| **Low to moderate mental health conditions** | | | | | | | | | | |
| --- | --- | --- | --- | --- | --- | --- | --- | --- | --- | --- |
| **By the end of year 1** | | | | | | | | | | |
|  | BCR | % of participants receiving IPS service that worked at any point in the first 12 months | | | | | | | | |
|  | **£1.33** | 48% | 50% | 52% | 55% | **57%** | 60% | 65% | 70% | 75% |
| IPS service cost per person (£) | £1,500 | £1.18 | £1.36 | £1.49 | £1.73 | £1.90 | £2.13 | £2.46 | £2.82 | £3.17 |
|  | £1,800 | £0.99 | £1.13 | £1.24 | £1.44 | £1.58 | £1.77 | £2.05 | £2.35 | £2.64 |
|  | £2,100 | £0.85 | £0.97 | £1.06 | £1.24 | £1.35 | £1.52 | £1.76 | £2.01 | £2.26 |
|  | **£2,326** | £0.76 | £0.88 | £0.96 | £1.12 | **£1.22** | £1.37 | £1.59 | £1.82 | £2.04 |
|  | £2,400 | £0.74 | £0.85 | £0.93 | £1.08 | £1.19 | £1.33 | £1.54 | £1.76 | £1.98 |
|  | £2,700 | £0.66 | £0.76 | £0.83 | £0.96 | £1.05 | £1.18 | £1.37 | £1.57 | £1.76 |
|  | £3,000 | £0.59 | £0.68 | £0.74 | £0.87 | £0.95 | £1.06 | £1.23 | £1.41 | £1.58 |
|  | £3,300 | £0.54 | £0.62 | £0.68 | £0.79 | £0.86 | £0.97 | £1.12 | £1.28 | £1.44 |
| **By the end of year 5** | | | | | | | | | | |
|  | BCR | % of participants receiving IPS service that worked at any point in the first 12 months | | | | | | | | |
|  | **£5.87** | 48% | 50% | 52% | 55% | **57%** | 60% | 65% | 70% | 75% |
| IPS service cost per person (£) | £1,500 | £7.12 | £7.40 | £7.67 | £8.32 | £8.80 | £9.60 | £10.77 | £12.02 | £13.19 |
|  | £1,800 | £5.93 | £6.17 | £6.39 | £6.93 | £7.33 | £8.00 | £8.98 | £10.02 | £10.99 |
|  | £2,100 | £5.09 | £5.29 | £5.48 | £5.94 | £6.29 | £6.85 | £7.70 | £8.59 | £9.42 |
|  | **£2,326** | £4.59 | £4.77 | £4.95 | £5.36 | **£5.67** | £6.19 | £6.95 | £7.75 | £8.50 |
|  | £2,400 | £4.45 | £4.63 | £4.80 | £5.20 | £5.50 | £6.00 | £6.73 | £7.51 | £8.24 |
|  | £2,700 | £3.96 | £4.11 | £4.26 | £4.62 | £4.89 | £5.33 | £5.99 | £6.68 | £7.33 |
|  | £3,000 | £3.56 | £3.70 | £3.84 | £4.16 | £4.40 | £4.80 | £5.39 | £6.01 | £6.59 |
|  | £3,300 | £3.24 | £3.36 | £3.49 | £3.78 | £4.00 | £4.36 | £4.90 | £5.46 | £5.99 |

Table S17 Example of Using IPS-MOD Tool to assess sensitivity of benefit-cost ratio to the IPS service cost per person - Low to moderate mental health conditions

| **Low to moderate mental health conditions AND low to moderate musculoskeletal conditions** | | | | | | | | | | |
| --- | --- | --- | --- | --- | --- | --- | --- | --- | --- | --- |
| **By the end of year 1** | | | | | | | | | | |
|  | BCR | % of participants receiving IPS service that worked at any point in the first 12 months | | | | | | | | |
|  | **£0.88** | 18% | 20% | 22% | 25% | **28%** | 30% | 35% | 40% | 45% |
| IPS service cost per person (£) | £1,500 | £0.63 | £0.73 | £0.84 | £1.06 | £1.19 | £1.31 | £1.57 | £1.88 | £2.16 |
|  | £1,800 | £0.52 | £0.61 | £0.70 | £0.88 | £0.99 | £1.09 | £1.31 | £1.57 | £1.80 |
|  | £2,100 | £0.45 | £0.52 | £0.60 | £0.75 | £0.85 | £0.94 | £1.12 | £1.34 | £1.54 |
|  | **£2,326** | £0.40 | £0.47 | £0.54 | £0.68 | **£0.77** | £0.84 | £1.02 | £1.21 | £1.39 |
|  | £2,400 | £0.39 | £0.46 | £0.52 | £0.66 | £0.74 | £0.82 | £0.98 | £1.17 | £1.35 |
|  | £2,700 | £0.35 | £0.41 | £0.46 | £0.59 | £0.66 | £0.73 | £0.87 | £1.04 | £1.20 |
|  | £3,000 | £0.31 | £0.36 | £0.42 | £0.53 | £0.60 | £0.65 | £0.79 | £0.94 | £1.08 |
|  | £3,300 | £0.28 | £0.33 | £0.38 | £0.48 | £0.54 | £0.60 | £0.72 | £0.85 | £0.98 |
| **By the end of year 5** | | | | | | | | | | |
|  | BCR | % of participants receiving IPS service that worked at any point in the first 12 months | | | | | | | | |
|  | **£5.03** | 18% | 20% | 22% | 25% | **28%** | 30% | 35% | 40% | 45% |
| IPS service cost per person (£) | £1,500 | £2.47 | £3.19 | £3.95 | £5.49 | £6.40 | £7.21 | £8.93 | £10.76 | £12.30 |
|  | £1,800 | £2.06 | £2.66 | £3.30 | £4.58 | £5.33 | £6.01 | £7.44 | £8.97 | £10.25 |
|  | £2,100 | £1.76 | £2.28 | £2.82 | £3.92 | £4.57 | £5.15 | £6.38 | £7.69 | £8.78 |
|  | **£2,326** | £1.59 | £2.06 | £2.55 | £3.54 | **£4.13** | £4.65 | £5.76 | £6.94 | £7.93 |
|  | £2,400 | £1.54 | £1.99 | £2.47 | £3.43 | £4.00 | £4.50 | £5.58 | £6.73 | £7.69 |
|  | £2,700 | £1.37 | £1.77 | £2.20 | £3.05 | £3.56 | £4.00 | £4.96 | £5.98 | £6.83 |
|  | £3,000 | £1.24 | £1.60 | £1.98 | £2.75 | £3.20 | £3.60 | £4.47 | £5.38 | £6.15 |
|  | £3,300 | £1.12 | £1.45 | £1.80 | £2.50 | £2.91 | £3.28 | £4.06 | £4.89 | £5.59 |

Table S18 Example of Using IPS-MOD Tool to assess sensitivity of benefit-cost ratio to the IPS service cost per person - Low to moderate mental health conditions AND low to moderate musculoskeletal
conditions

|  | **BCR By the end of year 5 alternative scenarios on duration of effect** | | |
| --- | --- | --- | --- |
|  | **MSK** | **MH** | **MSK+MH** |
| **Base case:** IPS full effect Year 1 then diminishes to 25% effect in Year 5 | £5.71 | £5.67 | £4.13 |
| **Alternative Scenario 1:** IPS full effect Year 1 then diminishes to Control in Year 5 | £5.58 | £5.56 | £4.02 |
| **Alternative Scenario 2:** IPS full effect until Year 2 then diminishes to Control in Year 5 | £5.76 | £5.72 | £4.18 |
| **Alternative Scenario 3:** IPS full effect until Year 3 then diminishes to Control in Year 5 | £5.89 | £5.84 | £4.30 |

Table S19 BCR By the end of year 5 alternative scenarios on duration of effect

Figure S9 Societal perspective Benefit Cost Ratio by Value of a QALY for Low to moderate musculoskeletal conditions

Figure S10 Societal perspective Benefit Cost Ratio by Value of a QALY for Low to moderate mental health conditions

Figure S11 Societal perspective Benefit Cost Ratio by Value of a QALY for Low to moderate mental health conditions AND low to moderate musculoskeletal conditions

## **5.4 Validating IPS-MOD against Health-Led Employment Trial Outcomes**

In this validation exercise, presented in Table S18, IPS-MOD was configured to match the health condition distribution and sickness-related benefit claim rates reported in the pooled “out of work” group from the HLET^6^. Specifically, IPS-MOD was set to the same proportions as HLET for:

- Participants with low-to-moderate musculoskeletal health conditions (11.53%)
- Participants with low-to-moderate mental health conditions (36.62%)
- Participants with both low-to-moderate mental and musculoskeletal conditions (51.85%)
- Participants claiming sickness-related benefits (11%).

Service costs were set at £2,326 per person, in line with the HLET costing, and the same value of a QALY was used, inflation-adjusted for 2023 (£79,467.99 for IPS-MOD compared with £69,494.02 in the HLET’s original 2020 values).

When calibrated to the HLET employment outcomes, IPS-MOD reproduced employment measures closely. For the control group, the proportion working at any point in year 1 was 31.04% in IPS-MOD versus 31.52% in HLET, with end-of-year employment at 19.32% versus 19.86%, respectively. For the intervention group, IPS-MOD estimated 36.49% working at any point in year 1 (HLET: 36.97%), with end-of-year employment at 23.93% (HLET: 23.55%). The proportion employed for at least 13 weeks was slightly lower in IPS-MOD than in HLET (control: 13.31% vs 18.00%; intervention: 16.94% vs 22%).

From an outcomes perspective, IPS-MOD predicted per-person QALY gains of 0.02 by the end of year 1 for the intervention group, consistent with HLET assumptions. Gains in average weeks of paid work were slightly higher in IPS-MOD than in the trial: 1.54 weeks in year 1 compared with 1.1 weeks in HLET, and 4.09 weeks by year 2 (22-month HLET measure) compared with 2.6 weeks in HLET.

Using the HLET’s published benefit–cost ratio (BCR) calculation method but substituting IPS-MOD outputs, the BCR by the end of year 1 was £1.12, close to the HLET-reported £1.22. This close alignment across employment outcomes and cost–benefit estimates provides reassurance that IPS-MOD can reliably replicate HLET results when configured with equivalent baseline characteristics and cost assumptions.

|  | IPS-MOD Control | HLET Published Results Control | IPS-MOD Intervention | HLET Published Results Intervention |
| --- | --- | --- | --- | --- |
| Baseline Characteristics | | | | |
| % Of participants with Low to moderate musculoskeletal health conditions | 11.53% | 11.53% | 11.53% | 11.53% |
| % Of participants with Low to moderate mental health conditions | 36.62% | 36.62% | 36.62% | 36.62% |
| % Of participants with Low to moderate mental health conditions AND low to moderate musculoskeletal conditions | 51.85% | 51.85% | 51.85% | 51.85% |
| % Of participants claiming sickness related benefits | 11% | 11% | 11% | 11% |
| Service cost (per person) | £2326 | £2326 | £2326 | £2326 |
| Value of a QALY | £70,000 (2021) | £60,000  (2020) | £70,000  (2021) | £60,000  (2020) |
| Value of a QALY (inflation adjusted to 2023) | £79,467.99 | £69,494.02 | £79,467.99 | £69,494.02 |
| Results | | | | |
| Non-employed at start | 100% | 100% | 100% | 100% |
| % Of individuals that worked any point in the first year | 31.04% | 31.52% | 36.49% | 36.97% |
| % of individuals employed by the end of year 1 | 19.32% | 19.86% | 23.93% | 23.55% |
| % of individuals who were employed for at least 13 weeks in the first year | 13.31% | 18.00% | 16.94% | 22% |
| QALY gains to those receiving IPS service (per person average) by the end of year 1 | - | - | 0.02 | 0.02 |
| Gains to average weeks in which paid work took place by the end of year 1 (per person average) | - | - | 1.54 | 1.1 |
| Gains to average weeks in which paid work took place by the end of year 2 (22-month measure for HLET) (per person average) | - | - | 4.09 | 2.6 |
| Societal perspective benefit cost ratio | | | | |
| By the end of year 1 | - | - | £1.12 | £1.22 |

Table S20 Validating IPS-MOD against Health-Led Employment Trial Outcomes

**References**

1	Department for Work and Pensions & Department of Health and Social Care. Health-led Employment Trial Evaluation: 12 month outcomes. 2023.

2	Westermann A, Fox S, Flowers J. fingertipsR: Fingertips data for public health. 2024.

3	Department for Work and Pensions, NatCen Social Research. Family resources survey. [Data series]. UK data service. SN: 200017. 2021. DOI:<http://doi.org/10.5255/UKDA-Series-200017>.

4	NatCen Social Research, University College London Department of Epidemiology and Public Health. Health survey for england, 2019. [Data collection]. UK data service. SN: 8860. 2023. DOI: [http://doi.org/10.5255/UKDA-SN-8860-1](%20http://doi.org/10.5255/UKDA-SN-8860-1).

5	Fujiwara D. The Department for Work and Pensions Social Cost-Benefit Analysis framework. Methodologies for estimating and incorporating the wider social and economic impacts of work in Cost-Benefit Analysis of employment programmes. *Department for Work and Pensions, Working Paper No 86* 2010.

6	Pensions DfWa. Health-led Employment Trial Evaluation 12-month outcomes report: Economic evaluation. 2022.

7	HM Treasury. The Green Book. 2022. <https://www.gov.uk/government/publications/the-green-book-appraisal-and-evaluation-in-central-government/the-green-book-2020>.

8	Gebel M, Voßemer J. [The impact of employment transitions on health in Germany. A difference-in-differences propensity score matching approach](https://doi.org/10.1016/j.socscimed.2014.02.039). *Social Science & Medicine* 2014; **108**: 128–36.

9	Devlin NJ, Shah KK, Feng Y, Mulhern B, Van Hout B. Valuing health-related quality of life: An EQ-5D-5L value set for england. *Health economics* 2018; **27**: 7–22.

10	Gray AM, Rivero-Arias O, Clarke PM. Estimating the association between SF-12 responses and EQ-5D utility values by response mapping. *Medical Decision Making* 2006; **26**: 18–29.

11	Morris D, Chen RKL. ukhlsclean: Data Wrangling for Understanding Society - the UK Household Longitudinal Study version 1.0.1. *University of Sheffield* 2024. DOI: [http://doi.org/10.17605/OSF.IO/6K9NV](%20http://doi.org/10.17605/OSF.IO/6K9NV).

12	Richiardi M, Collado D, Popova D. UKMOD - a new tax-benefit model for the four nations of the UK. *International Journal of Microsimulation* 2021; **14**: 92–101.

13	Morris D. frsclean: An R package for cleaning the Family Resources Survey version 1.0.2. *University of Sheffield* 2024. DOI: [http://doi.org/10.17605/OSF.IO/GNSEJ](%20http://doi.org/10.17605/OSF.IO/GNSEJ).
